# Assisted Reproductive Technology Surveillance — United States, 2015

**DOI:** 10.15585/mmwr.ss6703a1

**Published:** 2018-02-16

**Authors:** Saswati Sunderam, Dmitry M. Kissin, Sara B. Crawford, Suzanne G. Folger, Sheree L. Boulet, Lee Warner, Wanda D. Barfield

**Affiliations:** 1 Division of Reproductive Health, National Center for Chronic Disease Prevention and Health Promotion, CDC

## Abstract

**Problem/Condition:**

Since the first U.S. infant conceived with assisted reproductive technology (ART) was born in 1981, both the use of ART and the number of fertility clinics providing ART services have increased steadily in the United States. ART includes fertility treatments in which eggs or embryos are handled in the laboratory (i.e., in vitro fertilization [IVF] and related procedures). Although the majority of infants conceived through ART are singletons, women who undergo ART procedures are more likely than women who conceive naturally to deliver multiple-birth infants. Multiple births pose substantial risks for both mothers and infants, including obstetric complications, preterm delivery (<37 weeks), and low birthweight (<2,500 g) infants. This report provides state-specific information for the United States (including the District of Columbia and Puerto Rico) on ART procedures performed in 2015 and compares birth outcomes that occurred in 2015 (resulting from ART procedures performed in 2014 and 2015) with outcomes for all infants born in the United States in 2015.

**Period Covered:**

2015.

**Description of System:**

In 1995, CDC began collecting data on ART procedures performed in fertility clinics in the United States as mandated by the Fertility Clinic Success Rate and Certification Act of 1992 (FCSRCA) (Public Law 102–493 [October 24, 1992]). Data are collected through the National ART Surveillance System, a web-based data collection system developed by CDC. This report includes data from 52 reporting areas (the 50 states, the District of Columbia, and Puerto Rico).

**Results:**

In 2015, a total of 182,111 ART procedures (range: 135 in Alaska to 23,198 in California) with the intent to transfer at least one embryo were performed in 464 U.S. fertility clinics and reported to CDC. These procedures resulted in 59,334 live-birth deliveries (range: 55 in Wyoming to 7,802 in California) and 71,152 infants born (range: 68 in Wyoming to 9,176 in California). Nationally, the number of ART procedures performed per 1 million women of reproductive age (15–44 years), a proxy measure of the ART utilization rate, was 2,832. ART use exceeded the national rate in 13 reporting areas (California, Connecticut, Delaware, the District of Columbia, Hawaii, Illinois, Maryland, Massachusetts, New Hampshire, New Jersey, New York, Rhode Island, and Virginia).

Nationally, among ART transfer procedures in patients using fresh embryos from their own eggs, the average number of embryos transferred increased with increasing age of the woman (1.6 among women aged <35 years, 1.8 among women aged 35–37 years, and 2.3 among women aged >37 years). Among women aged <35 years, the national elective single-embryo transfer (eSET) rate was 34.7% (range: 11.3% in Puerto Rico to 88.1% in Delaware).

In 2015, ART contributed to 1.7% of all infants born in the United States (range: 0.3% in Puerto Rico to 4.5% in Massachusetts). ART also contributed to 17.0% of all multiple-birth infants, 16.8% of all twin infants, and 22.2% of all triplets and higher-order infants. The percentage of multiple-birth infants was higher among infants conceived with ART (35.3%) than among all infants born in the total birth population (3.4%). Approximately 34.0% of ART-conceived infants were twins and 1.0% were triplets and higher-order infants.

Nationally, infants conceived with ART contributed to 5.1% of all low birthweight infants. Among ART-conceived infants, 25.5% had low birthweight, compared with 8.1% among all infants. ART-conceived infants contributed to 5.3% of all preterm (gestational age <37 weeks) infants. The percentage of preterm births was higher among infants conceived with ART (31.2%) than among all infants born in the total birth population (9.7%). Among singletons, the percentage of ART-conceived infants who had low birthweight was 8.7% compared with 6.4% among all infants born. The percentage of ART-conceived infants who were born preterm was 13.4% among singletons compared with 7.9% among all infants.

**Interpretation:**

Multiple births from ART contributed to a substantial proportion of all twins, triplets, and higher-order infants born in the United States. For women aged <35 years, who are typically considered good candidates for eSET, the national average of 1.6 embryos was transferred per ART procedure. Of the four states (Illinois, Massachusetts, New Jersey, and Rhode Island) with comprehensive mandated health insurance coverage for ART procedures (i.e., coverage for at least four cycles of IVF), three (Illinois, Massachusetts, and New Jersey) had rates of ART use exceeding 1.5 times the national rate. This type of mandated insurance coverage has been associated with greater use of ART and likely accounts for some of the difference in per capita ART use observed among states.

**Public Health Action:**

Twins account for the majority of ART-conceived multiple births. Reducing the number of embryos transferred and increasing use of eSET when clinically appropriate could help reduce multiple births and related adverse health consequences for both mothers and infants. State-based surveillance of ART might be useful for monitoring and evaluating maternal and infant health outcomes of ART in states with high ART use.

## Introduction

Since the birth of the first U.S. infant conceived with assisted reproductive technology (ART) in 1981, use of advanced technologies to overcome infertility has increased, as has the number of fertility clinics providing ART services and procedures in the United States (*1*). In 1992, Congress passed the Fertility Clinic Success Rate and Certification Act (FCSRCA) (Public Law 102–493 [October 24, 1992]), which requires that all U.S. fertility clinics performing ART procedures report data to CDC annually on every ART procedure performed. CDC initiated data collection in 1995 and in 1997 published the first annual ART Fertility Clinic Success Rates Report (*2*). Two reports are now produced annually — ART Fertility Clinic Success Rates Report and ART National Summary Report (*1*,*3*) — and present several measures of success for ART, including the percentage of ART procedures and transfers that result in pregnancies, live-birth deliveries, singleton live-birth deliveries, and multiple live-birth deliveries.

Although ART helps millions of infertile couples to achieve pregnancy, ART is associated with potential health risks for both mothers and infants. Because multiple embryos are transferred in most ART procedures, ART often results in multiple-gestation pregnancies and multiple births (*4*–*11*). Risks to the mother from a multiple birth include higher rate of caesarean delivery, maternal hemorrhage, pregnancy-related hypertension, and gestational diabetes (*12*,*13*). Risks to the infant include prematurity, low birthweight, death, and greater risk for birth defects and developmental disability (*4*–*17*). Further, singleton infants conceived with ART might have higher risk for low birthweight and prematurity than singletons not conceived with ART (*18*). However, this higher risk might be associated with singleton births resulting from the transfer of more than one embryo in ART patients who are not good candidates for elective single-embryo transfer (eSET) (*19*).

This report was compiled on the basis of ART surveillance data reported to CDC’s Division of Reproductive Health for procedures performed in 2015. Data on the use of ART are presented for residents of each U.S. state, the District of Columbia, and Puerto Rico; data also are reported on outcomes for infants born in 2015 resulting from ART procedures performed in 2014 and 2015. The report also examines the contribution of ART to select outcomes (i.e., multiple-birth infants, low birthweight infants, and preterm infants) and compares outcomes among ART-conceived infants with outcomes among all infants born in the United States in 2015.

## Methods

### National ART Surveillance System

In 1995, CDC initiated data collection of ART procedures performed in the United States. ART data are obtained from all fertility clinics in the United States through the National ART Surveillance System (NASS), a web-based data collection system developed by CDC (https://www.cdc.gov/art/nass/index.html). Clinics that are members of the Society for Assisted Reproductive Technology (SART) can report their data to NASS through SART. Clinics that are not members of SART can enter their data directly into NASS. All clinics must verify the accuracy of their data that are reported in the clinic table in the annual ART Fertility Clinic Success Rates Report before finalizing submission of their data in NASS. The data then are compiled by Westat and reviewed by both CDC and Westat. A small proportion of clinics (7%) did not report their data to CDC in 2015 and are listed as nonreporting programs in the 2015 ART Fertility Clinic Success Rates Report, as required by FCSRCA. Because nonreporting clinics tend to be smaller on average than reporting clinics, NASS is estimated to contain information on 98% of all ART procedures in the United States (*1*).

Data collected include patient demographics, medical history, and infertility diagnoses; clinical information pertaining to the ART procedure type; and information regarding resultant pregnancies and births. The data file contains one record per ART procedure (or cycle of treatment) performed. Because ART providers typically do not provide continued prenatal care after a pregnancy is established, information on live births for all procedures is collected by ART clinics. In 2015, this information was obtained either directly from the patient (73.2%) or from the patient’s obstetric provider (25.7%) and reported to NASS. In 2015, approximately 1.1% of pregnancy outcomes were missing in NASS.

### ART Procedures

ART includes fertility treatments in which eggs or embryos are handled in a laboratory (i.e., in vitro fertilization [IVF], gamete intrafallopian transfer, and zygote intrafallopian transfer). More than 99% of ART procedures performed are IVF. Because an ART procedure consists of several steps over an interval of approximately 2 weeks, a procedure often is referred to as a cycle of treatment. An ART cycle usually begins with drug-induced ovarian stimulation. If eggs are produced, the cycle progresses to the egg-retrieval stage, which involves surgical removal of the eggs from the ovaries. After the eggs are retrieved, they are combined with sperm in the laboratory during the IVF procedure. For some IVF procedures (69% in 2015), a specialized technique (intracytoplasmic sperm injection) is used where a single sperm is injected directly into the egg. If successful fertilization occurs, the most viable embryos (i.e., those that appear morphologically most likely to develop and implant) are selected for transfer back into the uterus. If an embryo implants in the uterus, a clinical pregnancy is diagnosed by the presence of a gestational sac detectable by ultrasound. Most pregnancies will progress to a live-birth delivery, defined as the delivery of one or more live-born infants; however, some result in pregnancy loss (*20*). ART does not include treatments in which only sperm are handled (i.e., intrauterine insemination) or procedures in which a woman is administered drugs to stimulate egg production without the intention of having eggs retrieved.

ART procedures are classified on the basis of the source of the egg (patient or donor) and the status of the eggs and embryos. Both fresh and thawed embryos can be derived from fresh or frozen eggs of the patient or donor. Patient and donor embryos can be created using sperm from a partner or donor. ART procedures involving fresh eggs and embryos include an egg-retrieval stage. ART procedures that use thawed eggs or embryos do not include egg retrieval because the eggs were retrieved during a previous procedure; either the eggs were frozen or the eggs were fertilized and the resultant embryos were frozen until the current procedure. An ART cycle can be discontinued at any step for medical reasons or by patient choice.

### Birth Data for United States

Data on the total number of live-birth and multiple-birth infants in each reporting area in 2015 were obtained from U.S. natality files (*21*,*22*). The natality online databases report counts of live births and multiple births occurring within the United States to residents and nonresidents. The data are derived from birth certificates.

### Variables and Definitions

Data on ART and outcomes from ART procedures are presented by patient’s residence (i.e., reporting area) at the time of treatment, which might not be the same as the location where the procedure was performed. If information on patient’s residence was missing (0.8% of procedures performed in 2015 and 1.1% of live-birth deliveries occurring in 2015), residence was assigned as the location where the procedure was performed. ART procedures performed in the United States among nonresidents are included in NASS data; however, they are excluded from certain calculations for which the exact denominators are not known. To protect confidentiality in the presentation of data in tables, cells with values of 1–4 for ART-conceived infants and with values of 0–9 for all infants are suppressed, as are data that can be used to derive these cell values; these values are included in the ART totals and in totals for all infants. In some cases as applicable, states are not identified when reporting ranges to protect confidentiality. Because of small numbers, ART data from U.S. territories (with the exception of Puerto Rico) are not included in this report. In addition, estimates derived from cell values <20 in the denominator have been suppressed because they are unstable, and estimates could not be calculated when the denominator was zero (e.g., preterm birth among triplets in reporting areas with no triplet births).

This report presents data on all procedures initiated with the intent to transfer at least one embryo with the exception of cycles using fresh embryos created from frozen eggs. The number of ART procedures performed per 1 million women of reproductive age (15–44 years) was calculated, and the resulting rate approximates the proportion of women of reproductive age who used ART in each reporting area. However, this proxy measure of ART use is only an approximation because some women who use ART fall outside the age range of 15–44 years (approximately 10% in 2015) and some women might have had more than one procedure during the reporting period.

A live-birth delivery was defined as the birth of one or more live-born infants. A singleton live-birth delivery was defined as a birth of only one infant who was born live. A multiple live-birth delivery was defined as a birth of two or more infants, at least one of whom was born live. Low birthweight was defined as <2,500 g and very low birthweight as <1,500 g. Gestational age for births among women who did not undergo ART procedures was calculated using a new standard for estimating the gestational age of the newborn. Since 2014, the new measure — obstetric estimate of gestation at delivery (OE) — replaced the measure based on the date of the last normal menstrual period (LMP) (*22*). Methods of calculating gestational age among women who underwent ART procedures have not changed. For births to women who underwent fresh ART procedures, gestational age was calculated by subtracting the date of egg retrieval from the birth date and adding 14 days. For births to women who underwent frozen embryo cycles or fresh ART procedures for which the date of retrieval was not available, gestational age was calculated by subtracting the date of embryo transfer from the birth date and adding 17 days (to account for an average of 3 days in embryo culture). Preterm delivery was defined as gestational age <37 weeks and very preterm delivery as gestational age <32 weeks (*23*).

Elective single-embryo transfer is a procedure in which one embryo, selected from a larger number of available embryos, is placed in the uterus, with extra embryos cryopreserved. Fresh transfer procedures in which only one embryo was transferred but no embryos were cryopreserved are considered single-embryo transfer but not considered eSET. In this report, percentage of eSET procedures and average number of embryos transferred were calculated for patients who used fresh embryos from their own eggs, in which at least one embryo was transferred. The rate of eSET was calculated by dividing the total number of transfer procedures in which only one embryo was transferred and one or more embryos were cryopreserved by the sum of the total number of single-embryo transfer procedures where extra embryos were cryopreserved plus the total number of transfer procedures in which more than one embryo was transferred. Transfer procedures in which only one embryo was transferred but no embryos were cryopreserved were excluded from the calculation of eSET percentage. The average number of embryos transferred by age group (<35 years, 35–37 years, and >37 years) was calculated by dividing the total number of embryos transferred by the total number of embryo-transfer procedures performed among that age group.

The contribution of ART to all infants born in a particular reporting area was used as a second measure of ART use. The contribution of ART to adverse birth outcomes (e.g., preterm or low birthweight infant) was calculated by dividing the total number of outcomes among ART-conceived infants by the total number of outcomes among all infants born.

The percentage of infants (ART conceived and all infants) born in a reporting area was calculated by plurality (singleton, multiple, twin, and triplet and higher-order birth) by dividing the number of infants (ART conceived and all infants) in each plurality group by the total number of infants born (ART conceived and all infants). The percentage of infants with low birthweight and preterm delivery was also calculated for each plurality group (singleton, twin, and triplet and higher-order births) for both ART-conceived infants and all infants by dividing the number of low birthweight or preterm infants in each plurality group by the total number of infants in that plurality group.

### Content of This Report

This report provides information on U.S. ART procedures performed in 2015 and compares outcomes for ART-conceived infants born in 2015 (resulting from ART procedures performed in 2014 and 2015) with outcomes for all infants born in 2015 in the United States and Puerto Rico. For each of these reporting areas, data are presented on the number of ART procedures and embryo transfers performed; the resulting number of pregnancies, live-birth deliveries (overall, singleton, and multiple), and live-born infants; and the number of ART procedures in relation to the number of women in the reproductive age group (15–44 years) (*24*).* Data are also presented on the number of embryo-transfer procedures performed, the average number of embryos transferred, and the percentage of eSET procedures performed among women who used fresh embryos from their own fresh eggs, by age group.

For each reporting area, the proportions of singleton and multiple-birth (including twin and triplet and higher-order multiple) infants resulting from ART are compared with the respective proportions among all infants born in that location in 2015. Infants born in a reporting area during that year include those who were conceived naturally and those resulting from ART and other infertility treatments. To accurately assess the proportion of ART births among overall U.S. births in 2015, ART births were aggregated from two reporting years: 1) infants conceived with ART procedures performed in 2014 and born in 2015 (70% of the live-birth deliveries reported to the ART surveillance system for 2015) and 2) infants conceived with ART procedures performed in 2015 and born in 2015 (30% of the live-birth deliveries reported to the ART surveillance system for 2015). The report presents the number and percentage of selected adverse perinatal outcomes (low birthweight, very low birthweight, preterm delivery, and very preterm delivery) among ART-conceived infants and all infants by plurality, as well as the contribution of ART to these outcomes.

## Results

### Overview of Fertility Clinics

**FIGURE 1:**
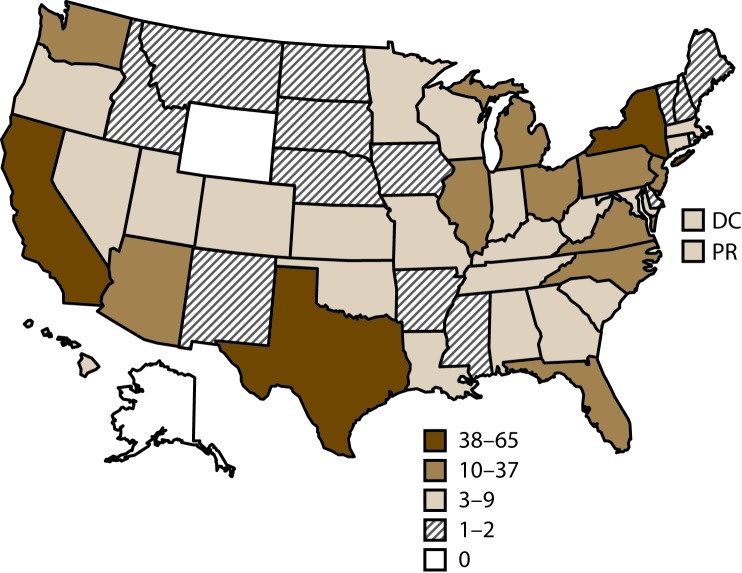
Location and number* of assisted reproductive technology clinics — United States and Puerto Rico, 2015 **Abbreviations:** DC = District of Columbia; PR = Puerto Rico. * In 2015, of the 499 clinics in the United States, 464 (93%) submitted data.

In 2015, of 499 fertility clinics in the United States that performed ART procedures, a total of 464 (93%) provided data to CDC, with the majority located in or near major cities (*1*). The number of fertility clinics performing ART procedures varied by reporting area. The reporting areas with the largest numbers of fertility clinics providing data were California (65), Texas (43), and New York (38) (Figure 1). 

### Number and Type of ART Procedures

The number, type, and outcomes of ART procedures performed are provided according to patient’s residence for all 52 reporting areas (Table 1). Residency data are missing for approximately 0.8% of procedures performed and 1.1% of live-birth deliveries; however, they are included in the totals. In 2015, approximately 16.3% of ART procedures were conducted in reporting areas other than the patient’s state of residence. Non-U.S. residents accounted for approximately 2.7% of ART procedures, 3.3% of ART live-birth deliveries, and 3.0% of ART-conceived infants born.

**TABLE 1:** Number* and outcomes of assisted reproductive technology procedures, by female patient’s reporting area of residence^†^ at time of treatment — United States and Puerto Rico, 2015

Patient’s reporting area of residence	No. of ART clinics^§^	No. of ART procedures performed	No. of ART embryo-transfer procedures^¶^	No. of ART pregnancies	No. of ART live-birth deliveries	No. of ART singleton live-birth deliveries	No. of ART multiple live-birth deliveries	No. of ART live-born infants	ART procedures per 1 million women aged 15–44 yrs**
Alabama	6	1,092	850	436	362	265	97	465	1,138.7
Alaska	0	135	111	70	60	41	19	80	921.4
Arizona^††^	12	2,393	1,959	1,052	823	630	193	1,026	1,813.0
Arkansas	1	528	417	190	167	124	43	209	915.9
California^††^	65	23,198	18,122	9,556	7,802	6,447	1,355	9,176	2,869.4
Colorado^††^	8	2,196	1,822	1,189	990	794	196	1,188	1,984.6
Connecticut	8	3,262	2,410	1,377	1,118	875	243	1,367	4,792.6
Delaware	2	714	508	306	255	231	24	279	3,948.9
District of Columbia	3	1,220	930	404	335	301	34	369	6,726.2
Florida	30	8,042	6,178	3,065	2,481	1,917	564	3,054	2,150.1
Georgia	8	3,904	3,153	1,656	1,316	1,123	193	1,515	1,841.6
Hawaii^††^	5	1,043	779	403	322	224	98	424	3,889.1
Idaho	1	543	453	271	227	171	56	285	1,708.6
Illinois	28	12,294	9,434	4,532	3,634	2,889	745	4,382	4,769.5
Indiana	10	2,144	1,733	822	678	507	171	855	1,653.7
Iowa	2	1,342	1,110	692	593	482	111	706	2,280.8
Kansas	4	1,040	761	410	339	271	68	409	1,856.7
Kentucky	5	1,238	1,041	508	409	316	93	503	1,453.7
Louisiana	5	1,425	972	507	411	315	96	508	1,509.6
Maine	1	452	383	186	157	136	21	178	1,947.3
Maryland	7	6,248	4,907	2,268	1,797	1,582	215	2,014	5,204.2
Massachusetts	8	9,388	7,866	3,626	2,911	2,579	332	3,241	6,832.8
Michigan	13	3,868	3,186	1,584	1,288	917	371	1,666	2,056.7
Minnesota	5	2,431	2,098	1,167	960	730	230	1,197	2,306.3
Mississippi	2	616	458	249	206	152	54	258	1,027.6
Missouri	8	2,254	1,843	916	776	593	183	963	1,915.8
Montana	1	293	230	132	109	75	34	143	1,567.5
Nebraska	2	751	573	323	275	212	63	338	2,049.8
Nevada	5	1,216	1,044	595	494	394	100	594	2,119.1
New Hampshire^††^	1	818	680	310	260	223	37	297	3,375.5
New Jersey	20	9,591	7,238	3,996	3,269	2,767	502	3768	5,580.7
New Mexico	1	215	170	91	73	59	14	88	543.6
New York	38	21,298	16,515	7,225	5,690	4,725	965	6,671	5,276.8
North Carolina	11	3,871	2,942	1,643	1,352	1,057	295	1,654	1,943.4
North Dakota	1	352	304	140	118	74	44	162	2,377.0
Ohio	14	4,250	3,589	1,811	1,498	1,102	396	1,903	1,925.0
Oklahoma	3	879	745	396	319	230	89	409	1,144.0
Oregon	3	1,261	994	619	524	394	130	657	1,603.6
Pennsylvania	16	6,683	5,310	2,445	1,991	1,658	333	2,326	2,783.6
Puerto Rico	3	229	206	99	71	46	25	97	329.3
Rhode Island	1	779	648	264	203	162	41	247	3,717.3
South Carolina	4	1,448	1,145	655	535	381	154	698	1,521.1
South Dakota	1	285	224	117	102	70	32	138	1,806.0
Tennessee	9	1,660	1,283	706	581	452	129	713	1,277.2
Texas	43	14,342	10,903	5,938	4,896	3,741	1,155	6,056	2,494.9
Utah^††^	4	1,838	1,585	954	794	565	229	1,023	2,821.9
Vermont	2	284	217	95	82	67	15	98	2,456.8
Virginia	12	5,966	4,755	2,265	1,838	1,545	293	2,133	3,540.9
Washington	12	3,445	2,706	1,470	1,202	1,013	189	1,395	2,430.8
West Virginia	3	305	253	118	104	76	28	134	914.6
Wisconsin	7	2,035	1,623	822	704	521	183	885	1,874.9
Wyoming	0	152	125	66	55	41	14	68	1,373.5
Nonresident	NA	4,855	3,795	2,133	1,778	1,423	355	2,140	—^§§^
**Total**	**464**	**182,111**	**143,286**	**72,870**	**59,334**	**47,685**	**11,649**	**71,152**	**2,832.1**

**Abbreviations:** ART = assisted reproductive technology; NA = not applicable.

* Excludes 45,779 egg/embryo-freezing and embryo-banking procedures, 4,003 egg-thaw procedures, and 43 procedures performed in territories not included in this report.

^†^ In cases of missing residency data (0.8%), the patient’s residence was assigned as the location in which the ART procedure was performed.

^§^ The ART procedures and outcomes by patient's reporting area of residence do not necessarily reflect the procedures and outcomes of the ART clinics within the reporting area, as some patients seek treatment at a clinic in a location other than their area of residence.

^¶^ Embryo-transfer procedures include all procedures performed in which an attempt was made to transfer at least one embryo.

** On the basis of U.S. Census Bureau estimates. **Source:** US Census Bureau. Annual estimates of the resident population for selected age groups by sex for the United States, states, counties, and Puerto Rico Commonwealth and municipios: April 1, 2010 to July 1, 2015. Washington, DC: US Census Bureau, Population Division; 2015. https://factfinder.census.gov/faces/tableservices/jsf/pages/productview.xhtml?pid=PEP_2015_PEPAGESEX&prodType=table.

^††^ In six states, >2% of residency information was missing for procedures performed: Arizona (4.7%), California (3.3%), Colorado (7.5%), Hawaii (5.4%), New Hampshire (3.5%), and Utah (5.8%). Overall, residency information was missing for 1,468 (0.8%) procedures performed and 623 (1.1%) live-birth deliveries.

^§§^ Non-U.S. residents excluded from rate because the appropriate denominators were not available.

**FIGURE 2:**
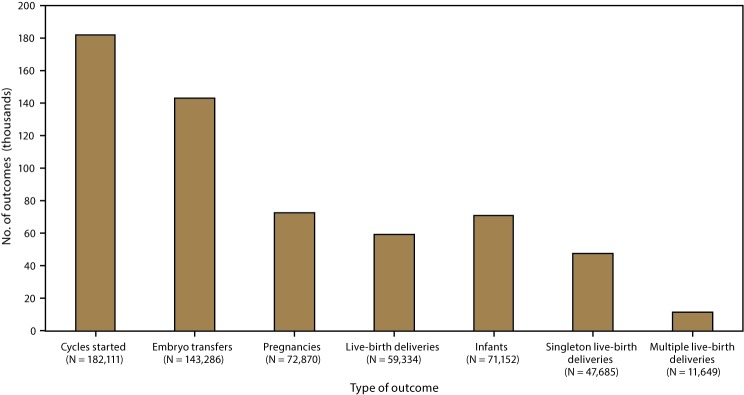
Number of outcomes of assisted reproductive technology procedures, by type of outcome — United States and Puerto Rico, 2015

In 2015, a total of 231,936 ART procedures were reported to CDC (*1*). Included in this report are data for 182,111 ART procedures performed in the United States (including Puerto Rico) with the intent to transfer at least one embryo. Excluded are 45,779 egg or embryo-freezing and embryo-banking procedures that did not result in an embryo transfer; 4,003 procedures started with the intent to thaw previously frozen eggs, fertilize the eggs, and then transfer the resulting fresh embryos; and 43 procedures that were performed in the territories not included in this report. Of 182,111 procedures performed in the reporting areas, a total of 143,286 (78.7%) progressed to embryo transfer (Table 1). Of 143,286 ART procedures that progressed to the embryo-transfer stage, 72,870 (50.9%) resulted in a pregnancy and 59,334 (41.4%) in a live-birth delivery. The 59,334 live-birth deliveries included 47,685 singleton live-birth deliveries (80.4%) and 11,649 multiple live-birth deliveries (19.6%) and resulted in 71,152 live-born infants (Table 1) (Figure 2).

Six reporting areas with the largest number of ART procedures (California, Illinois, Massachusetts, New Jersey, New York, and Texas) accounted for approximately half (49.5%) (90,111 of 182,111) of all ART procedures, 48.9% (70,078 of 143,286) of all embryo-transfer procedures, 46.8% (33,294 of 71,152) of all infants born who were conceived with ART, and 43.4% (5,054 of 11,649) of all ART-conceived multiple live-birth deliveries in the United States (Table 1). However, these six reporting areas accounted for only 36.6% of all U.S. births (*24*).

**FIGURE 3:**
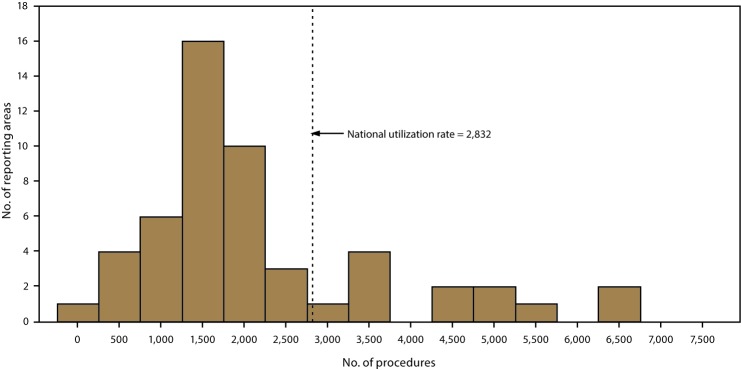
Number of reporting areas, by number of assisted reproductive technology procedures performed among women of reproductive age (15–44 years)* — United States and Puerto Rico, 2015 * Per 1 million women aged 15–44 years.

The number of ART procedures per 1 million women of reproductive age (15–44 years) varied (range: 329 in Puerto Rico to 6,833 in Massachusetts), with an overall national rate of 2,832. Thirteen reporting areas (California, Connecticut, Delaware, Hawaii, Illinois, Maryland, Massachusetts, New Hampshire, New Jersey, New York, Rhode Island, Virginia, and the District of Columbia) had ART use rates higher than the national rate. Of these reporting areas, Massachusetts (6,833) and the District of Columbia (6,726) had rates exceeding twice the national rate, while Connecticut (4,793), Illinois (4,770), Maryland (5,204), New Jersey (5,581), and New York (5,277) had rates exceeding 1.5 times the national rate (Table 1) (Figure 3).

### Embryo Transfer and Patient’s Age

The number of embryo-transfer procedures performed, the average number of embryos transferred per procedure, and the percentage of eSET procedures performed among women who used fresh embryos from their own fresh eggs are provided by reporting area and age group (Table 2). Overall, 27,942 embryo-transfer procedures were performed among women aged <35 years, 12,943 among women aged 35–37 years, and 18,429 among women aged >37 years. Nationally, on average, 1.6 embryos were transferred per procedure among women aged <35 years (range: 1.1 in Delaware to 2.0 in Puerto Rico), 1.8 among women aged 35–37 years (range: 1.5 in Delaware, Maine, Maryland, and Massachusetts to 2.2 in Puerto Rico), and 2.3 among women aged >37 years (range: 1.8 in Arkansas and Nevada to 2.8 in Rhode Island). Nationally, the percentage of eSET was 34.7% among women aged <35 years (range: 11.3% in Puerto Rico to 88.1% in Delaware), 20.8% among women aged 35–37 years (range: 0% in Puerto Rico to 61.9% in Delaware), and 2.3% among women aged >37 years (range: 0% in several reporting areas to 14.3% in Delaware). Among women aged <35 years, eSET percentage exceeded 1.5 times the national percentage in six reporting areas (Delaware, the District of Columbia, Maine, Massachusetts, Maryland, and New Hampshire).

**TABLE 2:** Number of assisted reproductive technology embryo-transfer procedures* among patients who used fresh embryos from their own fresh eggs, by female patient’s age group and reporting area of residence^†^ at time of treatment — United States and Puerto Rico, 2015

Patient’s reporting area of residence	<35 yrs			35–37 yrs			>37 yrs		
	No. of embryo-transfer procedures	Average no. of embryos transferred	eSET^§^ (%)	No. of embryo-transfer procedures	Average no. of embryos transferred	eSET (%)	No. of embryo-transfer procedures	Average no. of embryos transferred	eSET (%)
Alabama	266	1.8	19.4	75	2.0	4.3	64	2.5	0
Alaska	16	—^¶^	—^¶^	19	—^¶^	—^¶^	10	—^¶^	—^¶^
Arizona**	325	1.8	20.2	117	2.0	9.6	164	2.4	4.3
Arkansas	158	1.8	13.1	44	1.9	8.1	20	1.8	7.7
California**	2,196	1.6	36.1	1,339	1.9	21.5	2,273	2.5	4.4
Colorado**	181	1.7	26.7	73	1.8	16.1	69	2.2	5.3
Connecticut	621	1.5	46.5	255	1.8	18.5	385	2.2	5.2
Delaware	67	1.1	88.1	22	1.5	61.9	23	2.3	14.3
District of Columbia	93	1.4	56.4	74	1.6	34.5	237	2.0	8.0
Florida	1,167	1.7	26.4	613	1.9	10.5	891	2.2	2.8
Georgia	603	1.6	39.7	274	1.7	22.3	292	2.4	4.8
Hawaii**	96	1.8	22.5	59	1.9	11.3	150	2.5	3.1
Idaho	132	1.7	21.3	34	1.7	21.4	15	—^¶^	—^¶^
Illinois	2,110	1.7	30.4	928	1.9	16.1	1,319	2.3	2.8
Indiana	562	1.8	16.1	169	1.9	7.9	157	2.3	3.0
Iowa	295	1.5	46.0	90	1.6	36.7	79	2.0	6.0
Kansas	144	1.6	36.6	52	1.7	21.7	48	2.2	2.3
Kentucky	349	1.8	25.2	124	2.0	6.8	78	2.4	2.9
Louisiana	203	1.8	17.7	63	1.9	12.5	66	2.1	10.5
Maine	105	1.2	72.2	42	1.5	47.2	61	1.9	10.4
Maryland	1,107	1.3	63.0	527	1.5	40.7	954	2.0	8.4
Massachusetts	1,683	1.3	70.3	996	1.5	47.7	1,606	2.4	9.5
Michigan	853	1.8	19.2	310	2.0	11.6	385	2.3	4.6
Minnesota	629	1.6	35.1	214	1.7	20.2	184	2.1	4.6
Mississippi	111	1.7	26.0	29	1.9	11.1	23	2.1	0
Missouri	517	1.8	13.6	156	1.9	8.5	146	2.6	2.4
Montana	57	1.6	26.5	23	1.8	11.1	17	—^¶^	—^¶^
Nebraska	146	1.7	22.2	38	1.8	6.1	40	2.2	5.7
Nevada	152	1.6	26.4	72	1.7	14.5	73	1.8	5.4
New Hampshire**	161	1.4	62.9	98	1.6	38.3	97	2.5	3.9
New Jersey	1,258	1.5	45.5	670	1.6	31.8	965	2.1	12.9
New Mexico	15	—^¶^	—^¶^	6	—^¶^	—^¶^	10	—^¶^	—^¶^
New York	2,798	1.6	36.3	1,598	1.9	18.7	3,246	2.3	5.5
North Carolina	599	1.6	36.0	256	1.9	13.1	315	2.3	3.0
North Dakota	112	1.8	17.2	30	1.9	7.1	17	—^¶^	—^¶^
Ohio	1,091	1.8	19.4	397	1.9	7.7	399	2.3	3.4
Oklahoma	289	1.8	13.0	89	2.0	8.6	83	2.3	4.0
Oregon	150	1.7	26.6	67	1.8	23.4	53	1.9	13.6
Pennsylvania	1,211	1.6	36.6	571	1.8	21.0	585	2.2	6.0
Puerto Rico	56	2.0	11.3	37	2.2	0	62	2.4	0
Rhode Island	159	1.5	45.5	110	1.8	18.5	127	2.8	0
South Carolina	208	1.7	23.1	107	2	7.0	97	2.4	2.3
South Dakota	71	1.7	21.5	15	—^¶^	—^¶^	8	—^¶^	—^¶^
Tennessee	272	1.7	27.5	108	1.9	9.5	103	2.2	1.2
Texas	1,817	1.7	25.3	723	1.9	10.3	881	2.2	2.7
Utah**	523	1.8	20.7	151	1.9	11.3	120	2.1	5.0
Vermont	48	1.8	26.1	31	1.7	34.5	34	2.2	10.0
Virginia	904	1.5	42.0	510	1.6	28.0	732	2.0	6.8
Washington	452	1.5	43.3	222	1.7	29.4	264	2.1	3.6
West Virginia	95	1.8	17.3	32	1.9	16.0	18	—^¶^	—^¶^
Wisconsin	378	1.6	35.6	129	1.9	12.1	106	2.2	4.5
Wyoming	32	1.8	16.1	10	—^¶^	—^¶^	10	—^¶^	—^¶^
Nonresident	299	1.6	34.4	145	1.7	20.2	268	2.2	7.4
**Total**	**27,942**	**1.6**	**34.7**	**12,943**	**1.8**	**20.8**	**18,429**	**2.3**	**2.3**

**Abbreviation:** eSET = elective single-embryo transfer.

* Includes all procedures in which at least one embryo was transferred.

^†^ In cases of missing residency data (0.8%), the patient's residence was assigned as the location in which the assisted reproductive technology procedure was performed.

^§^ A procedure in which one embryo, selected from a larger number of available embryos, is placed in the uterus. A cycle in which only one embryo is available is not defined as eSET.

^¶^ Estimates on the basis of N <20 in the denominator have been suppressed because such rates are considered unstable.

** In six states, >2% of residency information was missing for procedures performed: Arizona (4.7%), California (3.3%), Colorado (7.5%), Hawaii (5.4%), New Hampshire (3.5%), and Utah (5.8%). Overall, residency information was missing for 1,468 (0.8%) procedures performed and 623 (1.1%) live-birth deliveries.

### Singleton and Multiple-Birth Infants

In 2015, among 4,009,654 infants born in the United States and Puerto Rico (*21*), a total of 66,298 (1.7%) were conceived with ART procedures performed in 2014 and 2015 (Table 3). California, Texas, and New York had the highest total number of all infants born (491,748, 403,618, and 237,274, respectively) and ART-conceived infants born (8,839, 5,778, and 6,435, respectively). The percentage of ART-conceived infants born among all infants born was highest in Massachusetts (4.5%), followed by the District of Columbia (3.7%), Connecticut (3.6%), and New Jersey (3.5%).

**TABLE 3:** Number, proportion, and percentage of infants born with use of assisted reproductive technology, by female patient’s reporting area of residence* at time of treatment — United States and Puerto Rico, 2015^†^

Patient’s reporting area of residence	Total no. of infants born^§^	No. of ART infants born	Proportion of ART infants among all infants (%)	Singleton infants among ART infants	Singleton infants among all infants^§^	Proportion of ART singleton infants among all singleton infants (%)
				No. (%)	No. (%)	
Alabama	**59,657**	435	0.7	250 (57.5)	57,444 (96.3)	0.4
Alaska	**11,282**	87	0.8	46 (52.9)	10,945 (97.0)	0.4
Arizona^¶^	**85,351**	962	1.1	576 (59.9)	82,823 (97.0)	0.7
Arkansas	**38,886**	217	0.6	124 (57.1)	37,624 (96.8)	0.3
California^¶^	**491,748**	8,839	1.8	5,853 (66.2)	476,094 (96.8)	1.2
Colorado^¶^	**66,581**	1,075	1.6	751 (69.9)	64,511 (96.9)	1.2
Connecticut	**35,746**	1,299	3.6	835 (64.3)	34,271 (95.9)	2.4
Delaware	**11,166**	262	2.3	214 (81.7)	10,798 (96.7)	2.0
District of Columbia	**9,578**	359	3.7	281 (78.3)	9,127 (95.3)	3.1
Florida	**224,269**	2,966	1.3	1,798 (60.6)	216,770 (96.7)	0.8
Georgia	**131,404**	1,582	1.2	1,089 (68.8)	126,872 (96.6)	0.9
Hawaii^¶^	**18,420**	506	2.7	274 (54.2)	17,900 (97.2)	1.5
Idaho	**22,827**	247	1.1	105 (42.5)	22,067 (96.7)	0.5
Illinois	**158,116**	4,056	2.6	2,551 (62.9)	151,956 (96.1)	1.7
Indiana	**84,040**	842	1.0	448 (53.2)	81,164 (96.6)	0.6
Iowa	**39,482**	646	1.6	417 (64.6)	38,136 (96.6)	1.1
Kansas	**39,154**	396	1.0	226 (57.1)	37,907 (96.8)	0.6
Kentucky	**55,971**	479	0.9	271 (56.6)	54,086 (96.6)	0.5
Louisiana	**64,692**	504	0.8	301 (59.7)	62,442 (96.5)	0.5
Maine	**12,607**	140	1.1	97 (69.3)	12,219 (96.9)	0.8
Maryland	**73,616**	1,891	2.6	1,468 (77.6)	71,026 (96.5)	2.1
Massachusetts	**71,492**	3,248	4.5	2,467 (76.0)	68,764 (96.2)	3.6
Michigan	**113,312**	1,474	1.3	859 (58.3)	109,084 (96.3)	0.8
Minnesota	**69,834**	1,109	1.6	684 (61.7)	67,355 (96.5)	1.0
Mississippi	**38,394**	260	0.7	137 (52.7)	37,023 (96.4)	0.4
Missouri	**75,061**	896	1.2	542 (60.5)	72,301 (96.3)	0.7
Montana	**12,583**	132	1.0	82 (62.1)	12,145 (96.5)	0.7
Nebraska	**26,679**	381	1.4	223 (58.5)	25,615 (96.0)	0.9
Nevada	**36,298**	531	1.5	332 (62.5)	35,099 (96.7)	0.9
New Hampshire^¶^	**12,433**	306	2.5	217 (70.9)	11,973 (96.3)	1.8
New Jersey	**103,127**	3,604	3.5	2,467 (68.5)	98,874 (95.9)	2.5
New Mexico	**25,816**	119	0.5	85 (71.4)	25,190 (97.6)	0.3
New York	**237,274**	6,435	2.7	4,367 (67.9)	228,485 (96.3)	1.9
North Carolina	**120,843**	1,564	1.3	994 (63.6)	116,592 (96.5)	0.9
North Dakota	**11,314**	138	1.2	80 (58.0)	10,963 (96.9)	0.7
Ohio	**139,264**	1,725	1.2	990 (57.4)	134,262 (96.4)	0.7
Oklahoma	**53,122**	415	0.8	223 (53.7)	51,412 (96.8)	0.4
Oregon	**45,655**	776	1.7	424 (54.6)	44,082 (96.6)	1.0
Pennsylvania	**141,047**	2,300	1.6	1,575 (68.5)	136,001 (96.4)	1.2
Puerto Rico	**31,157**	101	0.3	45 (44.6)	30,434 (97.7)	0.1
Rhode Island	**10,993**	268	2.4	174 (64.9)	10,590 (96.3)	1.6
South Carolina	**58,139**	611	1.1	358 (58.6)	56,011 (96.3)	0.6
South Dakota	**12,336**	104	0.8	74 (71.2)	11,946 (96.8)	0.6
Tennessee	**81,685**	694	0.8	428 (61.7)	78,875 (96.6)	0.5
Texas	**403,618**	5,778	1.4	3,445 (59.6)	390,594 (96.8)	0.9
Utah^¶^	**50,778**	954	1.9	504 (52.8)	48,940 (96.4)	1.0
Vermont	**5,903**	69	1.2	49 (71.0)	5,700 (96.6)	0.9
Virginia	**103,303**	2,055	2.0	1,492 (72.6)	99,693 (96.5)	1.5
Washington	**88,990**	1,390	1.6	980 (70.5)	86,107 (96.8)	1.1
West Virginia	**19,805**	143	0.7	82 (57.3)	19,198 (96.9)	0.4
Wisconsin	**67,041**	847	1.3	488 (57.6)	64,669 (96.5)	0.8
Wyoming	**7,765**	81	1.0	43 (53.1)	7,494 (96.5)	0.6
**Total**	**4,009,654**	**66,298**	**1.7**	**42,885 (64.7)**	**3,871,653 (96.6)**	**1.1**

**Abbreviation:** ART = assisted reproductive technology.

* In cases of missing residency data (0.8%), the patient's residence was assigned as the location in which the ART procedure was performed.

^†^ Includes infants conceived from ART procedures performed in 2014 and born in 2015 and infants conceived from ART procedures performed in 2015 and born in 2015. Total ART births exclude nonresidents.

^§^ U.S. births include nonresidents**. Source:** Martin JA, Hamilton BE, Osterman MJ, Driscoll AK, Mathews TJ. Births: final data for 2015. Natl Vital Stat Rep 2017;66:1–70.

^¶^ In six states, >2% of residency information was missing for procedures performed: Arizona (4.7%), California (3.3%), Colorado (7.5%), Hawaii (5.4%), New Hampshire (3.5%), and Utah (5.8%). Overall, residency information was missing for 1,468 (0.8%) procedures performed and 623 (1.1%) live-birth deliveries.

Nationally, 35.3% of ART-conceived infants were born in multiple-birth deliveries (range: 18.3% in Delaware to 57.5% in Idaho), compared with 3.4% of all infants (range: 2.3% in Puerto Rico to 4.7% in the District of Columbia) (Table 4). ART-conceived twins accounted for approximately 96.1% (22,491 of 23,413) of all ART-conceived infants born in multiple deliveries. ART-conceived multiple-birth infants contributed to 17.0% of all multiple-birth infants (range: 5.4% in New Mexico to 44.6% in Hawaii). Approximately 33.9% of all ART-conceived infants were twins (range: 16.0% in Delaware to 53.8% in Idaho), compared with 3.3% of all infants (range: 2.3% in Puerto Rico to 4.6% in the District of Columbia). ART-conceived twins contributed to 16.8% of all twins (range: 5.6% in New Mexico to 43.6% in Hawaii). Finally, 1.4% of ART-conceived infants were triplets and higher-order multiples (range: 0% in several states to 10.3% in Alaska), compared with 0.1%–0.2% of all infants. ART-conceived triplets and higher-order infants contributed to 22.2% of all triplets and higher-order infants (range: 0% in several states to 70.0% in Hawaii).

**TABLE 4:** Number, percentage, and proportion of multiple-birth infants, twins, and triplets and higher-order infants born with use of assisted reproductive technology procedures, by female patient’s reporting area of residence* at time of treatment — United States and Puerto Rico, 2015^†^

Patient’s reporting area of residence	Multiple-birth infants among ART infants^§^	Multiple-birth infants among all infants^¶^	Proportion of ART multiple-birth infants among all multiple-birth infants (%)	Twin infants among ART infants^§^	Twin infants among all infants^¶^	Proportion of ART twin infants among all twin infants (%)	Triplets and higher-order infants among ART infants^§^	Triplets and higher-order infants among all infants^¶^	Proportion of ART triplets and higher-order infants among all triplets and higher-order infants (%)
	No. (%)	No. (%)		No. (%)	No. (%)		No. (%)	No. (%)	
Alabama	185 (42.5)	2,213 (3.7)	8.4	169 (38.9)	2,110 (3.5)	8.0	16 (3.7)	103 (0.2)	15.5
Alaska	41 (47.1)	— (—)**	—**	—** (36.8)	328 (2.9)	9.8	9 (10.3)	— (—)**	—^††^
Arizona^¶¶^	386 (40.1)	2,528 (3.0)	15.3	356 (37.0)	2,444 (2.9)	14.6	30 (3.1)	8 (0.1)	35.7
Arkansas	93 (42.9)	1,262 (3.2)	7.4	— (—)**	1,223 (3.1)	—**	— (—)**	39 (0.1)	—**
California^¶¶^	2,986 (33.8)	15,654 (3.2)	19.1	2,893 (32.7)	15,250 (3.1)	19.0	93 (1.1)	404 (0.1)	23.0
Colorado^¶¶^	324 (30.1)	2,070 (3.1)	15.7	— (—)**	2,037 (3.1)	—**	— (—)**	33 (0)	—**
Connecticut	464 (35.7)	1,475 (4.1)	31.5	440 (33.9)	1,427 (4.0)	30.8	24 (1.8)	48 (0.1)	50.0
Delaware	48 (18.3)	368 (3.3)	13.0	42 (16.0)	353 (3.2)	11.9	6 (2.3)	15 (0.1)	—^††^
District of Columbia	78 (21.7)	451 (4.7)	17.3	78 (21.7)	438 (4.6)	17.8	0 (0)	13 (0.1)	—^††^
Florida	1,168 (39.4)	7,499 (3.3)	15.6	1,120 (37.8)	7,299 (3.3)	15.3	48 (1.6)	200 (0.1)	24.0
Georgia	493 (31.2)	4,532 (3.4)	10.9	460 (29.1)	4,391 (3.3)	10.5	33 (2.1)	141 (0.1)	23.4
Hawaii^¶¶^	232 (45.8)	520 (2.8)	44.6	218 (43.1)	500 (2.7)	43.6	14 (2.8)	20 (0.1)	70.0
Idaho	142 (57.5)	760 (3.3)	18.7	133 (53.8)	738 (3.2)	18.0	9 (3.6)	22 (0.1)	40.9
Illinois	1,505 (37.1)	6,160 (3.9)	24.4	1,451 (35.8)	5,999 (3.8)	24.2	54 (1.3)	161 (0.1)	33.5
Indiana	394 (46.8)	2,876 (3.4)	13.7	376 (44.7)	2,792 (3.3)	13.5	18 (2.1)	84 (0.1)	21.4
Iowa	229 (35.4)	1,346 (3.4)	17.0	224 (34.7)	1,307 (3.3)	17.1	5 (0.8)	39 (0.1)	12.8
Kansas	170 (42.9)	1,247 (3.2)	13.6	160 (40.4)	1,206 (3.1)	13.3	10 (2.5)	41 (0.1)	24.4
Kentucky	208 (43.4)	1,885 (3.4)	11.0	201 (42.0)	1,838 (3.3)	10.9	7 (1.5)	47 (0.1)	14.9
Louisiana	203 (40.3)	2,250 (3.5)	9.0	189 (37.5)	2,149 (3.3)	8.8	14 (2.8)	101 (0.2)	13.9
Maine	43 (30.7)	388 (3.1)	11.1	— (—)**	375 (3.0)	—**	— (—)**	13 (0.1)	—**^,††^
Maryland	423 (22.4)	2,590 (3.5)	16.3	405 (21.4)	2,518 (3.4)	16.1	18 (1.0)	72 (0.1)	25.0
Massachusetts	781 (24.0)	2,728 (3.8)	28.6	766 (23.6)	2,671 (3.7)	28.7	15 (0.5)	57 (0.1)	26.3
Michigan	615 (41.7)	4,228 (3.7)	14.5	588 (39.9)	4,068 (3.6)	14.5	27 (1.8)	160 (0.1)	16.9
Minnesota	425 (38.3)	2,479 (3.5)	17.1	404 (36.4)	2,379 (3.4)	17.0	21 (1.9)	100 (0.1)	21.0
Mississippi	123 (47.3)	1,371 (3.6)	9.0	123 (47.3)	1,353 (3.5)	9.1	0 (0)	18 (0)	—^††^
Missouri	354 (39.5)	— (—)**	12.8	330 (36.8)	2,677 (3.6)	12.3	24 (2.7)	83 (0.1)	28.9
Montana	50 (37.9)	— (—)**	—**	50 (37.9)	432 (3.4)	11.6	0 (0)	— (—)**	—^††^
Nebraska	158 (41.5)	1,064 (4.0)	14.8	— (—)**	1,018 (3.8)	—**	— (—)**	46 (0.2)	—**
Nevada	199 (37.5)	1,199 (3.3)	16.6	190 (35.8)	1,177 (3.2)	16.1	9 (1.7)	22 (0.1)	40.9
New Hampshire^¶¶^	89 (29.1)	460 (3.7)	19.3	— (—)**	448 (3.6)	—**	— (—)**	12 (0.1)	—**^,††^
New Jersey	1,137 (31.5)	4,253 (4.1)	26.7	1,112 (30.9)	4,138 (4.0)	26.9	25 (0.7)	115 (0.1)	21.7
New Mexico	34 (28.6)	626 (2.4)	5.4	34 (28.6)	610 (2.4)	5.6	0 (0)	16 (0.1)	—^††^
New York	2,068 (32.1)	8,789 (3.7)	23.5	2,019 (31.4)	8,500 (3.6)	23.8	49 (0.8)	289 (0.1)	17.0
North Carolina	570 (36.4)	4,251 (3.5)	13.4	552 (35.3)	4,120 (3.4)	13.4	18 (1.2)	131 (0.1)	13.7
North Dakota	58 (42.0)	— (—)**	—**	58 (42.0)	345 (3.0)	16.8	0 (0)	— (—)**	—^††^
Ohio	735 (42.6)	5,002 (3.6)	14.7	686 (39.8)	4,790 (3.4)	14.3	49 (2.8)	212 (0.2)	23.1
Oklahoma	192 (46.3)	1,710 (3.2)	11.2	— (—)**	1,682 (3.2)	—**	— (—)**	28 (0.1)	—**
Oregon	352 (45.4)	1,573 (3.4)	22.4	331 (42.7)	1,534 (3.4)	21.6	21 (2.7)	39 (0.1)	53.8
Pennsylvania	725 (31.5)	5,046 (3.6)	14.4	711 (30.9)	4,932 (3.5)	14.4	14 (0.6)	114 (0.1)	12.3
Puerto Rico	56 (55.4)	723 (2.3)	7.7	48 (47.5)	702 (2.3)	6.8	8 (7.9)	21 (0.1)	38.1
Rhode Island	94 (35.1)	403 (3.7)	23.3	88 (32.8)	382 (3.5)	23.0	6 (2.2)	21 (0.2)	28.6
South Carolina	253 (41.4)	2,128 (3.7)	11.9	238 (39.0)	2,085 (3.6)	11.4	15 (2.5)	43 (0.1)	34.9
South Dakota	30 (28.8)	390 (3.2)	7.7	24 (23.1)	374 (3.0)	6.4	6 (5.8)	16 (0.1)	—^††^
Tennessee	266 (38.3)	2,810 (3.4)	9.5	254 (36.6)	2,710 (3.3)	9.4	12 (1.7)	100 (0.1)	12.0
Texas	2,333 (40.4)	13,024 (3.2)	17.9	2,220 (38.4)	12,559 (3.1)	17.7	113 (2.0)	465 (0.1)	24.3
Utah^¶¶^	450 (47.2)	1,838 (3.6)	24.5	432 (45.3)	1,774 (3.5)	24.4	18 (1.9)	64 (0.1)	28.1
Vermont	20 (29.0)	— (—)**	—**	20 (29.0)	200 (3.4)	10.0	0 (0)	— (—)**	—^††^
Virginia	563 (27.4)	3,610 (3.5)	15.6	551 (26.8)	3,498 (3.4)	15.8	12 (0.6)	112 (0.1)	10.7
Washington	410 (29.5)	2,883 (3.2)	14.2	404 (29.1)	2,822 (3.2)	14.3	6 (0.4)	61 (0.1)	9.8
West Virginia	61 (42.7)	607 (3.1)	10.0	54 (37.8)	591 (3.0)	9.1	7 (4.9)	16 (0.1)	—^††^
Wisconsin	359 (42.4)	2,372 (3.5)	15.1	339 (40.0)	2,305 (3.4)	14.7	20 (2.4)	67 (0.1)	29.9
Wyoming	38 (46.9)	271 (3.5)	14.0	— (—)**	259 (3.3)	—**	— (—)**	12 (0.2)	—**^,††^
**Total**	**23,413 (35.3)**	**138,001 (3.4)**	**17.0**	**22,491 (33.9)**	**133,857 (3.3)**	**16.8**	**922 (1.4)**	**4,144 (0.1)**	**22.2**

**Abbreviation:** ART = assisted reproductive technology.

* In cases of missing residency data (0.8%), the patient’s residence was assigned as the location in which the ART procedure was performed.

^†^ ART totals include infants conceived from ART procedures performed in 2014 and born in 2015 and infants conceived from ART procedures performed in 2015 and born in 2015. Total ART births exclude nonresidents.

^§^ Includes only the number of infants live born in a multiple-birth delivery. For example, if three infants were born in a live-birth delivery and one of the three infants was stillborn, the total number of live-born infants would be two. However, the two infants still would be counted as triplets.

^¶^ U.S. births include nonresidents. **Source:** Martin JA, Hamilton BE, Osterman MJ, Driscoll AK, Mathews TJ. Births: final data for 2015. Natl Vital Stat Rep 2017;66:1–70.

** To protect confidentiality, cells with values of 1–4 for ART infants and cells with values of 0–9 for all infants are suppressed. Also suppressed are data that can be used to derive suppressed cell values. These values are included in the totals.

^††^ Estimates on the basis of N <20 in the denominator have been suppressed because such rates are considered unstable.

^¶¶^ In six states, >2% of residency information was missing for procedures performed: Arizona (4.7%), California (3.3%), Colorado (7.5%), Hawaii (5.4%), New Hampshire (3.5%), and Utah (5.8%). Overall, residency information was missing for 1,468 (0.8%) procedures performed and 623 (1.1%) live-birth deliveries.

### Adverse Perinatal Outcomes

Nationally, ART-conceived infants contributed to approximately 5.1% of all infants with low birthweight (range: 1.5% in New Mexico and Puerto Rico to 12.1% in Connecticut) and 5.0% of all infants with very low birthweight (range: 0% in South Dakota and Vermont to 10.9% in Massachusetts and North Dakota) (Table 5). In four reporting areas (Connecticut, Hawaii, Massachusetts, and New Jersey), >10% of all infants with low birthweight born were conceived with ART. In all reporting areas, the percentage of infants with low birthweight and very low birthweight was higher among those conceived with ART than among all infants. Among ART-conceived infants, 25.5% had low birthweight (range: 13.0% in Vermont to 48.5% in Puerto Rico), compared with 8.1% among all infants (range: 5.7% in Alaska to 11.4% in Mississippi). Approximately 4.3% of ART-conceived infants had very low birthweight (range: 0% in Vermont and South Dakota to 12.9% in Puerto Rico), compared with 1.4% among all infants (range: 0.8% in Montana to 2.2% in the District of Columbia).

**TABLE 5:** Number, percentage, and proportion of infants born with use of assisted reproductive technology,* by low birthweight category and female patient’s reporting area of residence^†^ at time of treatment — United States and Puerto Rico, 2015

Patient’s reporting area of residence	<2,500 g (LBW)			<1,500 g (VLBW)		
	ART infants	All infants^§^	Proportion of ART LBW infants among all LBW infants (%)	ART infants	All infants^§^	Proportion of ART VLBW infants among all VLBW infants (%)
	No. (%)	No. (%)		No. (%)	No. (%)	
Alabama	151 (35.1)	6,218 (10.4)	2.4	21 (4.9)	1,176 (2.0)	1.8
Alaska	24 (27.9)	646 (5.7)	3.7	— (—)^¶^	98 (0.9)	—^¶^
Arizona**	281 (29.6)	6,128 (7.2)	4.6	34 (3.6)	961 (1.1)	3.5
Arkansas	56 (25.9)	3,564 (9.2)	1.6	5 (2.3)	609 (1.6)	0.8
California**	2,113 (24.5)	33,666 (6.8)	6.3	322 (3.7)	5,527 (1.1)	5.8
Colorado**	268 (25.2)	6,001 (9.0)	4.5	32 (3.0)	761 (1.1)	4.2
Connecticut	342 (26.5)	2,836 (7.9)	12.1	50 (3.9)	557 (1.6)	9.0
Delaware	49 (18.8)	1,036 (9.3)	4.7	12 (4.6)	208 (1.9)	5.8
District of Columbia	60 (16.7)	959 (10.0)	6.3	10 (2.8)	206 (2.2)	4.9
Florida	781 (26.9)	19,306 (8.6)	4.0	151 (5.2)	3,433 (1.5)	4.4
Georgia	396 (25.2)	12,464 (9.5)	3.2	66 (4.2)	2,354 (1.8)	2.8
Hawaii**	163 (33.3)	1,531 (8.3)	10.6	26 (5.3)	245 (1.3)	10.6
Idaho	93 (37.7)	1,501 (6.6)	6.2	15 (6.1)	238 (1.0)	6.3
Illinois	1,030 (25.7)	13069 (8.3)	7.9	180 (4.5)	2,319 (1.5)	7.8
Indiana	241 (28.9)	6,725 (8.0)	3.6	59 (7.1)	1,209 (1.4)	4.9
Iowa	157 (24.3)	2,663 (6.7)	5.9	37 (5.7)	486 (1.2)	7.6
Kansas	116 (29.9)	2,672 (6.8)	4.3	16 (4.1)	476 (1.2)	3.4
Kentucky	122 (26.5)	4,846 (8.7)	2.5	11 (2.4)	784 (1.4)	1.4
Louisiana	158 (31.3)	6,839 (10.6)	2.3	34 (6.7)	1,261 (1.9)	2.7
Maine	27 (20.1)	871 (6.9)	3.1	— (—)^¶^	154 (1.2)	—^¶^
Maryland	386 (20.5)	6,297 (8.6)	6.1	72 (3.8)	1,202 (1.6)	6.0
Massachusetts	615 (19.2)	5,312 (7.4)	11.6	93 (2.9)	851 (1.2)	10.9
Michigan	401 (27.5)	9,612 (8.5)	4.2	77 (5.3)	1,707 (1.5)	4.5
Minnesota	275 (24.9)	4,494 (6.4)	6.1	42 (3.8)	799 (1.1)	5.3
Mississippi	81 (31.4)	4,387 (11.4)	1.8	19 (7.4)	817 (2.1)	2.3
Missouri	237 (28.2)	6,248 (8.3)	3.8	47 (5.6)	1,114 (1.5)	4.2
Montana	28 (21.2)	878 (7.0)	3.2	— (—)^¶^	95 (0.8)	—^¶^
Nebraska	104 (27.3)	1,893 (7.1)	5.5	10 (2.6)	293 (1.1)	3.4
Nevada	144 (28.6)	3,093 (8.5)	4.7	27 (5.4)	478 (1.3)	5.6
New Hampshire**	64 (21.1)	845 (6.8)	7.6	7 (2.3)	107 (0.9)	6.5
New Jersey	849 (23.7)	8,345 (8.1)	10.2	139 (3.9)	1,468 (1.4)	9.5
New Mexico	34 (28.8)	2,244 (8.7)	1.5	8 (6.8)	302 (1.2)	2.6
New York	1,510 (24.1)	18,507 (7.8)	8.2	251 (4.0)	3,188 (1.3)	7.9
North Carolina	405 (25.9)	11,023 (9.1)	3.7	63 (4.0)	2,106 (1.7)	3.0
North Dakota	36 (26.5)	700 (6.2)	5.1	15 (11.0)	138 (1.2)	10.9
Ohio	483 (28.2)	11,807 (8.5)	4.1	58 (3.4)	2,032 (1.5)	2.9
Oklahoma	133 (32.4)	4,172 (7.9)	3.2	17 (4.1)	726 (1.4)	2.3
Oregon	221 (28.7)	2,919 (6.4)	7.6	28 (3.6)	453 (1.0)	6.2
Pennsylvania	546 (24.1)	11,453 (8.1)	4.8	93 (4.1)	1,997 (1.4)	4.7
Puerto Rico	49 (48.5)	3,282 (10.5)	1.5	13 (12.9)	448 (1.4)	2.9
Rhode Island	53 (20.0)	833 (7.6)	6.4	10 (3.8)	155 (1.4)	6.5
South Carolina	173 (28.6)	5,535 (9.5)	3.1	32 (5.3)	1,029 (1.8)	3.1
South Dakota	24 (23.1)	754 (6.1)	3.2	0 (0)	127 (1.0)	0
Tennessee	179 (26.1)	7,460 (9.1)	2.4	35 (5.1)	1,318 (1.6)	2.7
Texas	1,690 (29.5)	33,275 (8.2)	5.1	352 (6.1)	5,683 (1.4)	6.2
Utah**	309 (32.6)	3,561 (7.0)	8.7	51 (5.4)	515 (1.0)	9.9
Vermont	9 (13.0)	386 (6.5)	2.3	0 (0)	51 (0.9)	0
Virginia	399 (19.6)	8,111 (7.9)	4.9	71 (3.5)	1,545 (1.5)	4.6
Washington	280 (20.3)	5,730 (6.4)	4.9	37 (2.7)	973 (1.1)	3.8
West Virginia	45 (31.7)	1,891 (9.5)	2.4	12 (8.5)	284 (1.4)	4.2
Wisconsin	235 (28.0)	4,870 (7.3)	4.8	49 (5.8)	868 (1.3)	5.6
Wyoming	25 (30.9)	666 (8.6)	3.8	— (—)^¶^	82 (1.1)	—^¶^
**Total**	**16,650 (25.5)**	**324,124 (8.1)**	**5.1**	**2,815 (4.3)**	**56,013 (1.4)**	**5.0**

**Abbreviations:** ART = assisted reproductive technology; LBW = low birthweight; VLBW = very low birthweight.

* ART totals include infants conceived from ART procedures performed in 2014 and born in 2015 and infants conceived from ART procedures performed in 2015 and born in 2015. Total ART infants exclude nonresidents and include only infants with birthweight data available.

^†^ In cases of missing residency data (0.8%), the patient’s residence was assigned as the location in which the ART procedure was performed.

^§^ U.S. births include nonresidents. **Source:** Martin JA, Hamilton BE, Osterman MJ, Driscoll AK, Mathews TJ. Births: final data for 2015. Natl Vital Stat Rep 2017;66:1–70.

^¶^ To protect confidentiality, cells with values of 1–4 for ART infants and cells with values of 0–9 for all infants are suppressed. Also suppressed are data that can be used to derive suppressed cell values. These values are included in the totals.

** In six states, >2% of residency information was missing for procedures performed: Arizona (4.7%), California (3.3%), Colorado (7.5%), Hawaii (5.4%), New Hampshire (3.5%), and Utah (5.8%). Overall, residency information was missing for 1,468 (0.8%) procedures performed and 623 (1.1%) live-birth deliveries.

Nationally, ART contributed to approximately 5.3% (range: 1.0% in Puerto Rico to 12.5% in Massachusetts) and 5.4% (range: 0% in Vermont to 11.9% in Massachusetts) of all infants born preterm and very preterm, respectively (Table 6). In four reporting areas (Connecticut, Hawaii, Massachusetts, and New Jersey), >10% of all infants born preterm and very preterm were conceived with ART. As with low birthweight, the percentage of infants who were born preterm and very preterm was higher among ART-conceived infants than among the total birth population. Among ART-conceived infants, 31.2% were born preterm (range: 11.6% in Vermont to 45.8% in Alabama), compared with 9.7% among all infants (range: 7.3% in Vermont to 15.0% in Puerto Rico). Approximately 5.2% of ART-conceived infants were born very preterm (range: 0% in Vermont to 14.9% in Puerto Rico), compared with 1.6% among all infants (range: 1.0% in Montana to 2.3% in Mississippi).

**TABLE 6:** Number, percentage, and proportion of infants born with use of assisted reproductive technology,* by preterm gestational age category and female patient’s reporting area of residence^†^ at time of treatment — United States and Puerto Rico, 2015

Patient’s reporting area of residence	<37 wks (PTB)			<32 wks (VPTB)		
	ART infants	All infants^§^	Proportion of ART PTB infants among all PTB infants (%)	ART infants	All infants^§^	Proportion of ART VPTB infants among all VPTB infants (%)
	No. (%)	No. (%)		No. (%)	No. (%)	
Alabama	198 (45.8)	7,544 (12.6)	2.6	38 (8.8)	1,268 (2.1)	3.0
Alaska	30 (34.9)	1,004 (8.9)	3.0	— (—)^¶^	130 (1.2)	—^¶^
Arizona**	359 (37.6)	7,724 (9.0)	4.6	59 (6.2)	1,121 (1.3)	5.3
Arkansas	66 (30.6)	4,201 (10.8)	1.6	8 (3.7)	683 (1.8)	1.2
California**	2,476 (28.3)	41,600 (8.5)	6.0	401 (4.6)	6,386 (1.3)	6.3
Colorado**	333 (31.1)	5,770 (8.7)	5.8	51 (4.8)	806 (1.2)	6.3
Connecticut	371 (28.6)	3,340 (9.3)	11.1	65 (5.0)	599 (1.7)	10.9
Delaware	65 (25.1)	1,093 (9.8)	5.9	12 (4.6)	219 (2.0)	5.5
District of Columbia	75 (21.1)	979 (10.2)	7.7	12 (3.4)	199 (2.1)	6.0
Florida	962 (32.6)	22,407 (10.0)	4.3	177 (6.0)	3,984 (1.8)	4.4
Georgia	526 (33.5)	14,133 (10.8)	3.7	80 (5.1)	2,567 (2.0)	3.1
Hawaii**	189 (37.4)	1,861 (10.1)	10.2	30 (5.9)	282 (1.5)	10.6
Idaho	100 (41.0)	1,852 (8.1)	5.4	9 (3.7)	255 (1.1)	3.5
Illinois	1,278 (31.7)	16,048 (10.1)	8.0	236 (5.9)	2,784 (1.8)	8.5
Indiana	318 (37.9)	8,061 (9.6)	3.9	68 (8.1)	1,349 (1.6)	5.0
Iowa	221 (34.2)	3,559 (9.0)	6.2	41 (6.3)	558 (1.4)	7.3
Kansas	140 (35.6)	3,426 (8.8)	4.1	17 (4.3)	526 (1.3)	3.2
Kentucky	186 (39.0)	6,026 (10.8)	3.1	21 (4.4)	870 (1.6)	2.4
Louisiana	223 (44.2)	7,964 (12.3)	2.8	38 (7.5)	1,414 (2.2)	2.7
Maine	43 (30.7)	1,062 (8.4)	4.0	8 (5.7)	187 (1.5)	4.3
Maryland	458 (24.3)	7,380 (10.0)	6.2	82 (4.4)	1,355 (1.8)	6.1
Massachusetts	749 (23.2)	6,002 (8.4)	12.5	119 (3.7)	999 (1.4)	11.9
Michigan	514 (34.9)	11,200 (9.9)	4.6	92 (6.3)	1,963 (1.7)	4.7
Minnesota	336 (30.4)	5,906 (8.5)	5.7	56 (5.1)	923 (1.3)	6.1
Mississippi	104 (40.0)	4,999 (13.0)	2.1	23 (8.8)	879 (2.3)	2.6
Missouri	321 (36.0)	7,504 (10.0)	4.3	59 (6.6)	1,246 (1.7)	4.7
Montana	32 (24.2)	1,058 (8.4)	3.0	— (—)^¶^	123 (1.0)	—^¶^
Nebraska	135 (35.4)	2,629 (9.9)	5.1	11 (2.9)	354 (1.3)	3.1
Nevada	163 (30.8)	3,604 (9.9)	4.5	24 (4.5)	542 (1.5)	4.4
New Hampshire**	67 (22.0)	977 (7.9)	6.9	12 (3.9)	146 (1.2)	8.2
New Jersey	1,080 (30.0)	10,064 (9.8)	10.7	166 (4.6)	1,630 (1.6)	10.2
New Mexico	44 (37.0)	2,459 (9.5)	1.8	10 (8.4)	332 (1.3)	3.0
New York	1,663 (25.9)	20,531 (8.7)	8.1	272 (4.2)	3,536 (1.5)	7.7
North Carolina	481 (30.9)	12,297 (10.2)	3.9	80 (5.1)	2,351 (1.9)	3.4
North Dakota	45 (32.8)	950 (8.4)	4.7	16 (11.7)	149 (1.3)	10.7
Ohio	581 (33.8)	14,300 (10.3)	4.1	67 (3.9)	2,393 (1.7)	2.8
Oklahoma	174 (42.0)	5,485 (10.3)	3.2	25 (6.0)	820 (1.5)	3.0
Oregon	255 (33.1)	3,459 (7.6)	7.4	41 (5.3)	521 (1.1)	7.9
Pennsylvania	671 (29.3)	13,224 (9.4)	5.1	99 (4.3)	2,257 (1.6)	4.4
Puerto Rico	45 (44.6)	4,663 (15.0)	1.0	15 (14.9)	698 (2.2)	2.1
Rhode Island	68 (25.4)	945 (8.6)	7.2	11 (4.1)	152 (1.4)	7.2
South Carolina	210 (34.7)	6,429 (11.1)	3.3	37 (6.1)	1,162 (2.0)	3.2
South Dakota	35 (34.3)	1,045 (8.5)	3.3	— (—)^¶^	131 (1.1)	—^¶^
Tennessee	252 (36.3)	8,959 (11.0)	2.8	43 (6.2)	1,443 (1.8)	3.0
Texas	2,234 (38.9)	41,019 (10.2)	5.4	414 (7.2)	6,470 (1.6)	6.4
Utah**	376 (39.5)	4,722 (9.3)	8.0	70 (7.4)	652 (1.3)	10.7
Vermont	8 (11.6)	429 (7.3)	1.9	0 (0)	70 (1.2)	0
Virginia	550 (26.8)	9,549 (9.2)	5.8	90 (4.4)	1,715 (1.7)	5.2
Washington	363 (26.2)	7,216 (8.1)	5.0	60 (4.3)	1,155 (1.3)	5.2
West Virginia	63 (44.4)	2,223 (11.2)	2.8	14 (9.9)	326 (1.6)	4.3
Wisconsin	300 (35.5)	6,271 (9.4)	4.8	58 (6.9)	1,001 (1.5)	5.8
Wyoming	29 (35.8)	762 (9.8)	3.8	— (—)^¶^	82 (1.1)	—^¶^
**Total**	**20,565 (31.2)**	**3,87,914 (9.7)**	**5.3**	**3,447 (5.2)**	**63,763 (1.6)**	**5.4**

**Abbreviations:** ART = assisted reproductive technology; PTB = preterm birth; VPTB = very preterm birth.

* ART totals include infants conceived from ART procedures performed in 2014 and born in 2015 and infants conceived from ART procedures performed in 2015 and born in 2015. Total ART births exclude nonresidents and include only infants with gestational age data available.

^†^ In cases of missing residency data (0.8%), the patient’s residence was assigned as the location in which the ART procedure was performed.

^§^ U.S. births include nonresidents. **Source:** Martin JA, Hamilton BE, Osterman MJ, Driscoll AK, Mathews TJ. Births: final data for 2015. Natl Vital Stat Rep 2017;66:1–70.

^¶^ To protect confidentiality, cells with values of 1–4 for ART infants and cells with values of 0–9 for all infants are suppressed. Also suppressed are data that can be used to derive suppressed cell values. These values are included in the totals.

** In six states, >2% of residency information was missing for procedures performed: Arizona (4.7%), California (3.3%), Colorado (7.5%), Hawaii (5.4%), New Hampshire (3.5%), and Utah (5.8%). Overall, residency information was missing for 1,468 (0.8%) procedures performed and 623 (1.1%) live-birth deliveries.

The percentage of ART-conceived infants who had low birthweight was 8.7% (range: 2.0% in one state to 22.2% in Puerto Rico) among singletons, 54.8% (range: 40.0% in Vermont to 70.8% in Puerto Rico) among twins, and 95.9% (range: 79.2% in Michigan to 100% in several reporting areas) among triplets and higher-order multiples; the corresponding percentage among all infants born was 6.4% (range: 4.6% in Alaska to 9.4% in Mississippi) among singletons, 55.2% (range: 40.5% in Alaska to 66.1% in Puerto Rico) among twins, and 95.0% (range: 76.2% in Puerto Rico to 100% in several states) among triplets and higher-order multiples (Table 7).

**TABLE 7:** Percentages of low birthweight infants (<2,500 g) among infants born with assisted reproductive technology* and all U.S. infants, by plurality and female patient’s reporting area of residence^†^ at time of treatment — United States and Puerto Rico, 2015

Patient’s reporting area of residence	ART singleton infants (%)	All singleton infants^§^ (%)	ART twin infants^¶^ (%)	All twin infants^§^ (%)	ART triplets and higher-order infants^¶^ (%)	All triplets and higher-order infants^§^ (%)
Alabama	10.1	8.3	66.5	63.8	—**	96.1
Alaska	—^††^	4.6	40.6	40.5	—**	—**^,††^
Arizona^¶¶^	9.1	5.7	58.3	52.8	96.2	96.4
Arkansas	5.7	7.4	51.1	61.3	—**^,††^	100.0
California^¶¶^	8.1	5.3	55.7	53.3	93.5	93.8
Colorado^¶¶^	9.4	7.3	61.5	63.3	—**^,††^	93.9
Connecticut	9.1	5.9	55.6	54.2	95.8	97.9
Delaware	8.9	7.6	57.1	52.7	—**^,††^	—**
District of Columbia	6.0	7.7	55.1	55.5	—^§§^	—**
Florida	9.3	6.9	52.2	57.2	100.0	96.5
Georgia^¶^	8.8	7.6	58.8	60.2	100.0	95.7
Hawaii^¶¶^	13.3	6.9	55.8	53.2	—**	100.0
Idaho	10.5	5.0	54.9	50.1	—**	100.0
Illinois	8.1	6.4	54.2	53.8	94.4	91.9
Indiana	7.0	6.3	51.6	54.3	—**	97.6
Iowa	9.4	5.0	50.4	54.8	—**	97.4
Kansas	8.6	5.3	56.3	51.2	—**	92.7
Kentucky	9.1	7.0	49.2	55.3	—**^,††^	100.0
Louisiana	8.6	8.6	62.4	64.2	—**	100.0
Maine	6.5	5.4	50.0	49.9	—**^,††^	—**
Maryland	9.1	6.8	58.1	55.0	—**	100.0
Massachusetts	7.9	5.6	55.0	53.1	—**	91.2
Michigan	9.7	6.6	51.6	54.6	79.2	88.8
Minnesota	7.9	4.8	50.5	48.7	95.2	88.0
Mississippi	9.5	9.4	56.2	64.0	—^§§^	—**
Missouri	8.8	6.4	54.9	57.3	100.0	96.4
Montana	8.5	5.4	42.0	50.5	—^§§^	—**^,††^
Nebraska	9.4	5.1	52.9	54.1	—**^,††^	87.0
Nevada	11.4	6.8	55.6	56.5	—**	81.8
New Hampshire^¶¶^	7.4	5.2	53.6	45.5	**^,††^	—**
New Jersey	9.4	6.1	54.0	52.9	100.0	93.0
New Mexico	15.5	7.3	61.8	62.3	—^§§^	—**
New York	9.1	6.0	54.5	54.2	98.0	96.9
North Carolina	9.9	7.4	52.5	54.6	—**	96.9
North Dakota	7.5	4.7	53.6	50.1	—^§§^	—**^,††^
Ohio	8.7	6.7	51.5	54.5	95.7	92.0
Oklahoma	8.1	6.3	59.9	55.1	—**^,††^	100.0
Oregon	8.3	4.9	50.5	47.5	100.0	100.0
Pennsylvania	8.9	6.4	57.0	53.2	—**	89.5
Puerto Rico	22.2	9.2	70.8	66.1	—**	76.2
Rhode Island	5.2	5.8	45.3	50.5	—**	95.2
South Carolina	6.0	7.7	57.6	57.6	—**	97.7
South Dakota	10.8	4.7	45.8	44.9	—**	—**
Tennessee	8.3	7.3	53.1	58.3	—**	98.0
Texas	9.3	6.5	57.3	58.1	95.6	96.8
Utah^¶¶^	9.6	5.2	57.2	54.2	—**	95.3
Vermont	—^††^	4.9	40.0	49.0	—^§§^	—**^,††^
Virginia	7.4	6.1	51.0	54.1	—**	92.9
Washington	7.4	5.0	50.8	49.4	—**	96.7
West Virginia	8.6	7.9	57.4	60.2	—**	—**
Wisconsin	7.0	5.6	54.0	52.0	100.0	95.5
Wyoming	11.6	6.6	48.6	57.5	—**^,††^	—**
**Total**	**8.7**	**6.4**	**54.8**	**55.2**	**95.9**	**95.0**

**Abbreviation:** ART = assisted reproductive technology.

* ART totals include infants conceived from ART procedures performed in 2014 and born in 2015 and infants conceived from ART procedures performed in 2015 and born in 2015. Total ART births exclude nonresidents and only include infants with birthweight data available.

^†^ In cases of missing residency data (0.8%), the patient’s residence was assigned as the location in which the ART procedure was performed.

^§^ U.S. births include nonresidents. **Source:** Martin JA, Hamilton BE, Osterman MJ, Driscoll AK, Mathews TJ. Births: final data for 2015. Natl Vital Stat Rep 2017;66:1–70.

^¶^ Includes only the number of infants live born in a multiple-birth delivery. For example, if three infants were born in a live-birth delivery and one of the three infants was stillborn, the total number of live-born infants would be two. However, the two infants still would be counted as triplets.

** Estimates on the basis of N <20 in the denominator have been suppressed because such rates are considered unstable.

^††^ To protect confidentiality, cells with values of 1–4 for ART infants, and cells with values of 0–9 for all infants are suppressed. Also suppressed are data that can be used to derive suppressed cell values. These values are included in the totals.

^§§^ Estimates not calculated because N = 0 for the denominator.

^¶¶^ In six states, >2% of residency information was missing for procedures performed: Arizona (4.7%), California (3.3%), Colorado (7.5%), Hawaii (5.4%), New Hampshire (3.5%), and Utah (5.8%). Overall, residency information was missing for 1,468 (0.8%) procedures performed and 623 (1.1%) live-birth deliveries.

The percentage of ART-conceived infants who were born preterm was 13.4% among singletons (range: 6.3% in North Dakota to 20.3% in South Dakota), 62.4% among twins (range: 30.0% in Vermont to 83.2% in Alabama), and 97.7% among triplets and higher-order infants (range: 87.1% in California to 100% in several reporting areas); the corresponding percentage among all infants was 7.9% for singletons (range: 5.5% in Vermont to 13.8% in Puerto Rico), 59.0% for twins (range: 47.0% in Alaska to 70.5% in Louisiana), and 98.6% for triplets and higher-order infants (range: 90.9% in Nevada to 100% in several reporting areas) (Table 8).

**TABLE 8:** Percentages of preterm (<37 weeks) infants among infants born with use of assisted reproductive technology* and all U.S. infants, by plurality and female patient’s reporting area of residence^†^ at time of treatment — United States and Puerto Rico, 2015

Patient’s reporting area of residence	ART singleton infants (%)	All singleton infants^§^ (%)	ART twin infants^¶^ (%)	All twin infants^§^ (%)	ART triplets and higher-order infants^¶^ (%)	All triplets and higher-order infants^§^ (%)
Alabama	17.3	9.6	83.2	66.4	—**	97.1
Alaska	11.1	7.7	50.0	47.0	—**	—**^,††^
Arizona^¶¶^	14.2	7.5	70.1	58.6	100.0	98.8
Arkansas	7.3	8.9	60.0	66.1	—**^,††^	100.0
California^¶¶^	12.0	6.9	59.3	55.1	87.1	99.0
Colorado^¶¶^	14.0	7.0	70.1	59.2	—**^,††^	100.0
Connecticut	12.9	7.3	54.5	54.6	100.0	100.0
Delaware	14.6	8.1	70.0	58.6	—**	—**
District of Columbia	9.0	8.0	64.1	53.4	—^§§^	—**
Florida	14.5	8.2	58.8	60.1	93.8	99.0
Georgia	14.7	8.9	73.3	62.1	100.0	97.9
Hawaii^¶¶^	12.1	8.5	65.1	60.4	—**	100.0
Idaho	13.3	6.4	60.2	58.0	—**	100.0
Illinois	13.7	8.1	60.9	59.9	100.0	98.8
Indiana	11.9	7.8	65.9	58.5	—**	100.0
Iowa	14.4	7.0	69.6	64.0	—**	97.4
Kansas	9.9	7.1	67.5	56.1	—**	100.0
Kentucky	15.9	8.9	68.3	63.0	—**	100.0
Louisiana	17.3	10.2	83.1	70.5	—**	98.0
Maine	14.4	6.8	66.7	56.8	—**^,††^	—**
Maryland	12.9	8.2	62.2	59.0	—**	100.0
Massachusetts	11.5	6.5	59.5	55.4	—**	100.0
Michigan	14.4	7.9	61.9	59.6	100.0	96.3
Minnesota	10.4	6.8	60.7	52.8	100.0	98.0
Mississippi	16.8	11.0	65.9	67.8	—^§§^	—**
Missouri	16.0	7.9	63.9	64.5	100.0	100.0
Montana	9.8	6.7	48.0	56.5	—^§§^	—**^,††^
Nebraska	15.7	7.5	62.6	63.7	—**^,††^	93.5
Nevada	13.9	8.2	56.8	59.3	—**	90.9
New Hampshire^¶¶^	8.3	6.2	53.5	49.6	—**^,††^	—**
New Jersey	15.4	7.7	60.9	56.5	100.0	99.1
New Mexico	18.8	8.2	82.4	63.6	—^§§^	—**
New York	12.1	6.9	54.0	53.9	93.9	99.3
North Carolina	13.7	8.4	60.0	57.1	—**	95.4
North Dakota	6.3	6.9	69.0	55.7	—^§§^	—**^,††^
Ohio	11.4	8.3	61.2	61.1	100.0	98.1
Oklahoma	18.9	8.6	68.3	61.9	—**^,††^	100.0
Oregon	11.2	6.0	56.8	50.1	100.0	100.0
Pennsylvania	14.1	7.5	61.7	58.7	—**	98.2
Puerto Rico	15.6	13.8	62.5	66.1	—**	90.5
Rhode Island	10.3	6.9	50.0	51.6	—**	100.0
South Carolina	12.1	9.1	64.4	60.8	—**	100.0
South Dakota	20.3	6.8	63.6	56.4	—**	—**
Tennessee	15.0	9.1	69.3	62.4	—**	100.0
Texas	16.1	8.4	71.5	62.3	97.3	98.7
Utah^¶¶^	15.1	7.3	65.3	61.6	—**	100.0
Vermont	—^††^	5.5	30.0	47.5	—^§§^	—**^,††^
Virginia	12.9	7.4	62.8	57.5	—**	95.5
Washington	12.0	6.5	59.4	54.9	—**	100.0
West Virginia	19.8	9.3	74.1	70.4	—**	—**
Wisconsin	13.8	7.6	62.8	56.9	100.0	100.0
Wyoming	16.3	7.8	54.3	62.5	—**^,††^	—**
**Total**	**13.4**	**7.9**	**62.4**	**59.0**	**97.7**	**98.6**

**Abbreviation:** ART = assisted reproductive technology.

* ART totals include infants conceived from ART procedures performed in 2014 and born in 2015 and infants conceived from ART procedures performed in 2015 and born in 2015. Total ART births exclude nonresidents and includes only infants with gestational age data available.

^†^ In cases of missing residency data (0.8 %), the patient's residence was assigned as the location in which the ART procedure was performed.

^§^ U.S. births include nonresidents. **Source:** Martin JA, Hamilton BE, Osterman MJ, Driscoll AK, Mathews TJ. Births: final data for 2015. Natl Vital Stat Rep 2017;66:1–70.

^¶^ Includes only the number of infants live born in a multiple-birth delivery. For example, if three infants were born in a live-birth delivery and one of the three infants was stillborn, the total number of live-born infants would be two. However, the two infants still would be counted as triplets.

** Estimates on the basis of N <20 in the denominator have been suppressed because such rates are considered unstable.

^††^ To protect confidentiality, cells with values of 1–4 for ART infants and cells with values of 0–9 for all infants are suppressed. Also suppressed are data that can be used to derive suppressed cell values. These values are included in the totals.

^§§^ Estimates not calculated because N = 0 for the denominator.

^¶¶^ In six states, >2% of residency information was missing for procedures performed: Arizona (4.7%), California (3.3%), Colorado (7.5%), Hawaii (5.4%), New Hampshire (3.5%), and Utah (5.8%). Overall, residency information was missing for 1,468 (0.8%) procedures performed and 623 (1.1%) live-birth deliveries.

## Discussion

### Overview

The use of ART has increased substantially in the United States since the beginning of ART surveillance. In 1996 (the first full year for which ART data were reported to CDC), a total of 20,597 infants were born from 64,036 ART procedures (*25*). Since then, the number of procedures reported to CDC and the number of infants born from ART procedures have approximately tripled. Several changes can be observed in ART use and outcomes since the preceding reporting year in 2014 (*26*). The rate of ART use as measured by procedures performed per 1 million women of reproductive age (15–44 years) increased from 2,647 in 2014 to 2,832 in 2015. Among women aged <35 years, the average number of embryos transferred remained at 1.6; however, the percentage of eSET increased from 28.5% to 34.7%. Overall, the percentage of ART-conceived twins decreased from 37.5% to 33.9%, and the percentage of ART-conceived triplets and higher-order infants decreased from 1.8% to 1.4%. The contribution of ART-conceived twins to all twins decreased from 18.0% to 16.8%. The contribution of ART-conceived infants to all triplets and higher-order infants decreased from 26.4% to 22.2%. However, the contribution of ART to rates of multiple births and poor birth outcomes remained substantial. In 2015, the multiple birth rate was 10 times higher among ART-conceived infants compared with all infants (35.3% versus 3.4%), and although infants conceived with ART accounted for approximately 1.7% of total births in the United States, the proportion of multiple-birth deliveries attributable to ART was 17.0%.

ART-conceived twins accounted for approximately 96.1% (22,491 of 23,413) of all ART-conceived infants born in multiple-birth deliveries. On average, 1.6 embryos were transferred among women aged <35 years, even though single-embryo transfers have been associated with better perinatal outcomes among the majority of women in this age group (*27**,**28*). The percentage of infants with low birthweight and born preterm was substantially higher among ART-conceived infants (25.5% and 31.2%, respectively) than among all infants (8.1% and 9.7%, respectively). Similar to births among the general population, ART-conceived twins and triplets and higher-order infants were more likely than singletons to be born preterm (4.7 times and seven times, respectively).

Comparable data on ART use and embryo transfer practices from 18 European countries indicate that in 2012, ART use as defined by the number of procedures performed per 1 million women of reproductive age was 6,525; this was approximately 2.6 times higher than the rate in the United States in 2012 (*29*,*30*). Percentages of single-embryo transfers (eSET rates are not reported) varied widely in Europe, and a few countries reported a single-embryo transfer rate of over 50%. Overall, in these 18 reporting countries, approximately 82.1% of all IVF deliveries were singleton deliveries, compared with 73.5% in the United States (*29*,*30*).

### Variations in ART Use by Reporting Area

ART use (as measured by the number of ART procedures performed per 1 million women of reproductive age) varied widely by reporting area: residents of California, Connecticut, Delaware, Hawaii, Illinois, Maryland, Massachusetts, New Hampshire, New Jersey, New York, Rhode Island, Virginia, and the District of Columbia had higher ART use than the national rate. Although some women who used ART might have been aged >44 years, the measure for women aged 15–44 years is still useful as a proxy indicator of all ART use in each reporting area. Residents of California, Illinois, Massachusetts, New Jersey, New York, and Texas accounted for 46.8% of all infants conceived with ART. The large number of ART procedures performed in these states is a result of the size of the general population (California and Texas), higher rates of ART use (Massachusetts and New Jersey), or both (New York and Illinois).

The contribution of ART to all infants born varied substantially, even among states with high ART use (range: 1.8% in California to 4.5% in Massachusetts). State-level differences might be explained in part by variations in health insurance coverage. Fifteen states (Arkansas, California, Connecticut, Hawaii, Illinois, Louisiana, Maryland, Massachusetts, Montana, New Jersey, New York, Ohio, Rhode Island, Texas, and West Virginia) have passed legislation mandating that private insurers provide coverage for some fertility treatments, although not all mandates require coverage for ART. Mandates from four of these states (Illinois, Massachusetts, New Jersey, and Rhode Island) include comprehensive coverage for at least four cycles of IVF.^†^ Three of the four states with comprehensive mandates (Illinois, Massachusetts, and New Jersey) had rates of ART use that were at least 50% higher than the national rate. Insurance mandates for infertility treatments have been associated with greater use of ART (*31*–*33*). In two states with insurance mandates (Massachusetts and New Jersey), the average number of embryos transferred was less than the national rate and the rate of eSET was higher than the national rate among patients aged <35 years.

### Elective Single-Embryo Transfer Rates

Recommendations issued by the American Society of Reproductive Medicine (ASRM) and SART on the number of embryos to transfer have been revised several times to reduce the likelihood of higher-order multiple deliveries (*34*–*38*). New guidance issued by ASRM and SART in 2017 is focused on promoting single-embryo transfer and reducing all multiple pregnancies, including twin gestations. Recommendations for single-embryo transfer are now expanded to patients of any age transferring an euploid (i.e., chromosomally normal) embryo, selected with the assistance of preimplantation genetic screening, and for patients aged <38 years with any one of these criteria: 1) availability of high-quality embryos for cryopreservation, 2) a history of success with IVF procedures, 3) availability of vitrified blastocyst stage embryos, or 4) undergoing their first frozen embryo transfer (*39*). Results of an analysis of ART cycles conducted in 2015 suggested that approximately half of ART-related multiple births resulted from the transfer of two fresh embryos among women aged <35 years and patients who received donor oocytes; therefore, reducing the number of embryos transferred from two to one among these patients who have a good chance of pregnancy and live birth with single-embryo transfers will lower rates of ART-conceived twins (*40*,*41*).

Among women aged <35 years, the percentage of eSET procedures was higher (34.7%) than among those in older age groups (20.8% among women aged 35–37 years and 2.3% among women aged >37 years) and varied widely among reporting areas (range: 6.7%–88.1%). From 2009 to 2015, the national percentage of eSET increased nearly fivefold (from 7.4% to 34.7%) among women aged <35 years (*26*). From 2014 to 2015, the national percentage of eSET among women aged <35 years increased from 28.5% to 34.7%. However, the percentage of eSET is still lower in the United States than in countries that impose restrictions on the number of embryos transferred and provide public funding for ART services (ranging from two to six publicly funded cycles in some countries) (*42*). The eSET rates might be influenced by factors such as the patient’s age and diagnosis, as well as treatment costs that are typically high and often paid out of pocket by the patient (*31*). In the United States, even where mandated, coverage for infertility treatment can vary in scope, with ART services often excluded or restricted to certain age groups or diagnoses (*31*). Furthermore, insurance mandates for infertility do not apply to employers that self-insure. In three of the four states with mandatory comprehensive insurance coverage for ART, the eSET percentage among women aged <35 years was higher than the national percentage of 34.7% (70.3% in Massachusetts, 45.5% in New Jersey, and 45.5% in Rhode Island). ART procedures are expensive; out-of-pocket costs per IVF attempt are estimated to be between $10,000 and $15,000 (*32*). Insurance mandates for infertility and enhanced coverage for ART might increase the use of eSET because patients might be more willing to transfer fewer embryos when the financial burden of treatment is diminished (*32*,*43*,*44*). In the United States, efforts to increase acceptance and use of eSET still have barriers. Improving adherence to professional guidance on embryo transfer practices along with expanded insurance coverage for ART services might promote greater use of eSET (*40*,*41*,*44*,*45*).

### ART Multiple-Birth Infants

Singleton live-birth deliveries have lower risks than multiple-birth deliveries for adverse birth outcomes such as prematurity, low birthweight, disability, and death (*46*–*48*). To optimize healthy birth outcomes, the transfer of fewer embryos should be encouraged where appropriate, taking into consideration the patient’s age and prognosis (*27*). The percentage of ART-conceived multiple-birth infants in the United States decreased from 53.1% in 2000 to 35.3% in 2015 (*49*). A substantial decrease was noted in both the percentage of ART-conceived triplets and higher-order infants (from 8.9% in 2000 to 1.4% in 2015) and the percentage of ART-conceived twins (from 44.2% in 2000 to 33.9% in 2015).

In the past, the slow decrease in twin-infant birth rates among women who undergo ART procedures was largely attributable to small but gradual increases in eSET rates (*40*,*41*). From 2013 to 2014, a historically large increase (33.0%) in the national eSET rate was observed (*26*). From 2014 to 2015, the increase in the national eSET rate was also substantial (21.8%) (*26*). Despite increased eSET use, ART-conceived twins still accounted for approximately one third of all ART-conceived infants in 2015, and on average, 1.6 embryos were transferred among patients aged <35 years. High rates of ART-conceived twins might be partially explained by the desire for more than one child among couples experiencing infertility and their perception that the benefits of a multiple-gestation pregnancy (compared with no pregnancy) outweigh the risks (*50*–*52*). Therefore, understanding the perspective of couples undergoing infertility treatments regarding multiple-gestation pregnancies and multiple births is important. The use and acceptance of eSET among younger patients with favorable prognoses might be improved through patient education (*53*,*54*). Patient education focusing on maternal and perinatal morbidity and mortality, and the economic costs of twin gestations, has been effective in reducing the preference for twins among patients (*53*–*55*).

The economic costs of multiple births also underscore the importance of efforts to reduce ART-related multiple births. In 2013, the mean health care cost to patients and insurers was estimated to be $26,922 for ART-conceived singleton deliveries, $115,238 for ART-conceived twins, and $434,668 for ART-conceived triplets and higher-order infants (*56*). Transferring two embryos is associated with a slight increase in overall birth rate but a greater increase in the twin birth rate compared with transferring a single embryo (*27*,*57*). However, transferring two embryos sequentially (single-embryo transfer over two sequential procedures) has similar cumulative live-birth rates and lower twin delivery rates than transferring two embryos in a single procedure and might be a cost-effective transfer approach, where estimated costs include ART treatment and pregnancy- and infant-associated medical costs (*58*–*60*). Evidence from other countries suggests that insurance coverage for ART combined with restrictions on the number of embryos transferred per cycle can reduce multiple births (*42*).

### ART Low Birthweight Infants and Preterm Births

The percentage of infants born preterm and very preterm was higher among ART-conceived infants than among infants in the total birth population. Four states (Connecticut, Hawaii, Massachusetts, and New Jersey) that had large numbers of ART procedures performed per 1 million women of reproductive age and a high proportion of ART-conceived infants born in the state also had high contribution of ART (>10%) to both categories of low birthweight and preterm births. In the United States, the contribution of ART to preterm births, the majority of which are also infants with low birthweight, is a key concern. Fertility treatments, both ART and controlled ovarian stimulations, contribute substantially to preterm births (*47*,*61*). Preterm births are a leading cause of infant mortality and morbidity; preterm infants are at increased risk for death and have more health and developmental problems than full-term infants (*47*,*62*–*64*). The health risks associated with preterm birth have contributed to increased health care costs. In 2005, the societal economic cost associated with all preterm births in the United States was estimated at $26 billion annually ($51,600 per infant born preterm) (*47*). In 2012, the societal economic cost associated with ART-conceived preterm infants in the United States was estimated at approximately $1.3 billion (*65*).

In addition to the known risks for multiple births associated with ART, even singleton infants conceived with ART procedures might be at increased risk for low birthweight and preterm delivery compared with infants born in the general population. However, a study published in 2017 found no significant differences in adverse outcomes among singleton infants conceived after single-embryo transfer among ART patients compared with singletons not conceived with ART, whereas singleton infants conceived after double-embryo transfer were more likely to have adverse perinatal outcomes. Those findings suggested that such differences might be attributable to the transfer of more than one embryo in patients who are not candidates for eSET and might have underlying pathologies (*19*).

## Limitations

The findings in this report are subject to at least five limitations. First, ART surveillance data were reported for each ART procedure performed rather than for each patient who used ART. As a result, because patients can achieve a successful pregnancy after undergoing multiple procedures, the procedure-specific success rates reported here underestimate the true per-patient success rates. Second, prematurity and low birthweight could be associated with factors contributing to underlying infertility or other maternal factors and not entirely to ART procedures. Third, approximately 7.0% of fertility clinics that performed ART in 2015 did not report their data to CDC, and these clinics might have had results differing from reporting clinics. Fourth, NASS lacks data on embryo quality, which influences the use of eSET among patients aged <35 years with favorable prognoses. Finally, in 2014 the methods for estimating gestational age for women who did not undergo ART changed from LMP measures to OE-based measures. The OE-based preterm birth rates are lower than those estimated with LMP, and therefore comparisons with previous years should be made with caution.

## Conclusion

Since 1995, the number of ART procedures performed in the United States and the number of infants born as a result of these procedures have nearly tripled. With this increasing use, ART-conceived infants represented 1.7% of infants born in the United States in 2015 and noticeably contributed to the prevalence of low birthweight and preterm deliveries, as approximately two fifths of ART-conceived infants were multiple-birth deliveries. Furthermore, among ART-conceived infants, although the percentage of triplets or higher-order infants has decreased since 2000, the percentage of twins has remained high. Because of the higher rates of preterm birth and low birthweight among multiple-birth infants, the impact of ART on poor birth outcomes remains substantial. This report documents the ART use rates and contribution of ART to multiple-birth deliveries, low birthweight, and preterm birth by patient’s reporting area of residence. This report also highlights the differences in percentage of low birthweight and prematurity between ART-conceived infants and all infants in the total birth population. These findings allow state health departments to monitor the extent of ART-related adverse perinatal outcomes among singletons, twins, and triplets and higher-order infants in their reporting areas.

Comprehensive insurance coverage of ART can help increase access to fertility treatments (*45*). Increased use of ART in reporting areas with insurance mandates also can result in higher absolute numbers of ART-conceived multiple-birth deliveries. The findings in this report indicate that ART use was higher than the national rate in all four states with statewide-mandated comprehensive insurance coverage. Three of these four states (Illinois, Massachusetts, and New Jersey) had use rates exceeding 1.5 times the national rate and two (Massachusetts and New Jersey) had a percentage of multiple births that was lower than the national percentage. Further, in both Massachusetts and New Jersey, among patients aged <35 years, the average number of embryos transferred was less than the national rate and the rate of eSET was higher than the national rate. More research is needed to ascertain the influence of state health insurance mandates on ART use, embryo transfer practices, infant outcomes, and economic and out-of-pocket patient costs of multiple births (*28*,*34*,*40*,*41*). Addressing the risk for multiple-birth deliveries also requires understanding the perspectives of couples undergoing infertility treatments who might view a multiple birth, especially twins, as an acceptable or desired outcome or who might lack awareness of the increased risks associated with multiple births to mothers and infants. Although the majority of clinicians acknowledge that the birth of a healthy singleton is the best outcome of ART, they might be sensitive to patient perspectives and experiences with infertility (*34*,*35*). Clinicians need to be aware of ongoing efforts and newly published guidance (*39*) to limit the number of embryos transferred to reduce the rate of multiple births, particularly twins. The wider implementation of eSET, when clinically appropriate, should be encouraged as a mechanism of promoting singleton infant births among ART pregnancies (*27*,*39*,*41*).

In 2014, CDC outlined a public health strategy for the detection, prevention, and management of infertility, including improving ART practice and outcomes, through coordinated efforts of government and nongovernment organizations. This national effort involves federal, state, and local agencies; the scientific community; health care professionals; insurance providers; employers; industry; nonprofit organizations; and organizations representing persons coping with infertility (*66*). Of public health importance is the role that infertility treatment has on adverse birth outcomes, primarily because of higher rates of multiple births. ART only partially explains the overall prevalence of these adverse outcomes in the United States. Other factors influencing multiple births include maternal age at conception and the use of non-ART fertility treatments (*47*,*61*,*67*). During 1980–2009, the older age of women giving birth accounted for a substantial increase in twins, thought to be attributed to the increased likelihood of an embryo splitting as a woman ages (*67*). The risk for multiple gestations associated with non-ART fertility treatments (i.e., controlled ovarian stimulation and ovulation induction coupled with timed intercourse or intrauterine insemination) is less well documented than that associated with ART procedures because clinics are only required to report data on ART use. However, research suggests that non-ART fertility treatments might contribute a larger percentage of multiple births than ART fertility treatments. In 2011, approximately 19% of twin births and 45% of triplet or higher-order births in the United States were attributable to non-IVF fertility treatments whereas 17% of twin births and 32% of triplet or higher-order births were attributable to IVF fertility treatments (*61*). Further efforts are needed to monitor the use of non-ART fertility treatments and their role in multiple-birth deliveries, particularly because the ability to control the occurrence of a multiple birth is more challenging when using non-ART fertility treatments (*47*,*61*). Multiple gestations resulting from non-ART fertility treatments also contribute to preterm births (*47*,*61*). Additional research is needed to identify the causes and consequences of preterm births that occur specifically as a result of infertility treatments and support further guidance to reduce the number of multiple gestations (*47*,*61*). However, studies have demonstrated that singleton infants conceived with ovulation stimulation are more likely than naturally conceived infants to be small for gestational age (*68*). CDC is monitoring the prevalence of non-ART fertility treatment use and resultant outcomes among women who had live births in several states participating in the Pregnancy Risk Assessment Monitoring System (*69*).

As of January 2016, all states have adopted the 2003 revision of the birth certificate that includes information on whether the pregnancy resulted from the use of infertility treatment; 47 states and the District of Columbia differentiate between the use of ART and non-ART treatments. CDC also is working to improve state-based surveillance of ART, infertility, and other birth-related matters by linking data from NASS to data collected by states (i.e., birth certificate, infant death, hospital discharge, and birth defect registry information). This initiative, the States Monitoring Assisted Reproductive Technology (SMART) Collaborative (https://www.cdc.gov/art/smart/index.html), has been determined to be feasible and useful for monitoring long-term outcomes of ART in selected states (*70**,**71*). CDC will continue to provide updates of ART use in the United States as data become available.

## Footnotes

* Data regarding population size were compiled on the basis of July 1, 2015, estimates from the U.S. Census Bureau.

† Eight states (Arkansas, Connecticut, Hawaii, Illinois, Maryland, Massachusetts, New Jersey, and Rhode Island) have insurance mandates that cover at least one ART cycle. Seven states (California, Louisiana, Montana, New York, Ohio, Texas, and West Virginia) have insurance mandates that exclude IVF coverage. Information is available at https://resolve.org/what-are-my-options/insurance-coverage/coverage-state/.
